# The Rho Exchange Factors Vav2 and Vav3 Favor Skin Tumor Initiation and Promotion by Engaging Extracellular Signaling Loops

**DOI:** 10.1371/journal.pbio.1001615

**Published:** 2013-07-23

**Authors:** Mauricio Menacho-Márquez, Ramón García-Escudero, Virginia Ojeda, Antonio Abad, Pilar Delgado, Clotilde Costa, Sergio Ruiz, Balbino Alarcón, Jesús M. Paramio, Xosé R. Bustelo

**Affiliations:** 1 Centro de Investigación del Cáncer, Consejo Superior de Investigaciones Científicas (CSIC)–University of Salamanca, Salamanca, Spain; 2 Instituto de Biología Molecular y Celular del Cáncer, Consejo Superior de Investigaciones Científicas (CSIC)–University of Salamanca, Salamanca, Spain; 3 Molecular Oncology Unit, Centro de Investigaciones Energéticas, Medioambientales y Tecnológicas, Madrid, Spain; 4 Centro de Biología Molecular “Severo Ochoa,” CSIC–Madrid Autonomous University, Madrid, Spain; Friedrich Miescher Institute, Switzerland

## Abstract

The catalytic activity of GDP/GTP exchange factors (GEFs) is considered critical to maintain the typically high activity of Rho GTPases found in cancer cells. However, the large number of them has made it difficult to pinpoint those playing proactive, nonredundant roles in tumors. In this work, we have investigated whether GEFs of the Vav subfamily exert such specific roles in skin cancer. Using genetically engineered mice, we show here that Vav2 and Vav3 favor cooperatively the initiation and promotion phases of skin tumors. Transcriptomal profiling and signaling experiments indicate such function is linked to the engagement of, and subsequent participation in, keratinocyte-based autocrine/paracrine programs that promote epidermal proliferation and recruitment of pro-inflammatory cells. This is a pathology-restricted mechanism because the loss of Vav proteins does not cause alterations in epidermal homeostasis. These results reveal a previously unknown Rho GEF-dependent pro-tumorigenic mechanism that influences the biology of cancer cells and their microenvironment. They also suggest that anti-Vav therapies may be of potential interest in skin tumor prevention and/or treatment.

## Abstract 2

Rho GEFs Vav2 and Vav3 regulate pro-tumorigenic autocrine/paracrine signals in keratinocytes and are dispensable for skin homeostasis.

## Author Summary

GDP/GTP exchange factors (GEFs) involved in Rho GTPase activation have been traditionally considered as potential anticancer drug targets. However, little is known about the best GEFs to inhibit in different tumor types, the pro-tumorigenic programs that they regulate, and the collateral effects that inactivation may induce in healthy tissues. Here, we investigate this issue in HRas-dependent skin tumors using genetic techniques. Despite the large number of Rho GEFs present in both normal and tumoral epidermis, we demonstrate that the co-expression of the exchange factors Vav2 and Vav3 is critical for the development of this tumor type. We also identify a previously unknown Vav-dependent autocrine/paracrine program that favors keratinocyte survival/proliferation and the formation of an inflammatory state during the initiation and promotion phases of this tumor. Finally, our results indicate that inactivation of Vav proteins is innocuous for the homeostasis of normal epidermis. Taken together, these results imply that Vav protein-based therapies may be of interest for skin tumor prevention and/or treatment.

## Introduction

Rho guanosine nucleotide exchange factors (Rho GEFs) promote the transition of Rho family GTP hydrolases (GTPases) from the inactive (GDP bound) to the active (GTP bound) state during signal transduction [1],[2]. These enzymes can be subdivided into the Dbl-homology (DH) and the Dedicator of Cytokinesis (Dock) families based on the catalytic domain utilized for the GDP/GTP exchange reaction. A common feature of these two families is their extreme diversity because, in mammals, they are composed of 54 and 11 members, respectively. These family members vary widely in terms of catalytic specificity, presence of regulatory and effector domains, mechanism of activation, and expression patterns. As a consequence, they are key elements to adapt the activation of Rho GTPases to specific cell types, membrane receptors, or subcellular localizations [1],[2]. Rho GEFs have been traditionally regarded as important for tumorigenesis and, thereby, as potential drug targets [3]. However, the large number of Rho GEFs and their regulatory complexity have made it difficult to identify which ones were the most important for the development and/or progression of specific tumors. Inferences from sequencing data have not been useful in this case, because their genes seldom undergo mutations in cancer cells [3]. The use of animal models in this functional context has been also rather limited. However, the few studies available do support the idea that these enzymes have pro-tumorigenic functions. Thus, the T-cell lymphoma invasion and metastasis-inducing protein 1 (Tiam1, ID number: 21844) has been shown to be important for both cutaneous squamous and colorectal [4],[5] tumors. The adenomatous polyposis coli-stimulated exchange factor 1 (Asef1, ID number: 226970) and Asef2 (ID number: 219140) proteins have been linked to colorectal cancer [6]. Finally, Vav3 (ID number: 57257) and phosphatidylinositol 3,4,5-triphosphate-dependent Rac exchanger 1 (P-Rex1, ID number: 277360) are involved in the development of p190^Brc/Abl^-driven acute lymphoblastic leukemia and melanoma, respectively [7],[8].

Vav proteins exemplify well the complexity existing in the large Rho GEF family. Thus, this subfamily has three members in vertebrates (Vav1 [ID number: 22324], Vav2 [ID number: 22325], Vav3) that display overlapping but not identical expression patterns. They all share similar structures that encompass a complex array of regulatory, catalytic, and protein–protein interaction domains [9]. These domains enable them to interact with and become activated by receptors with either intrinsic or associated tyrosine kinase activity, activate GDP/GTP exchange on Rho GTPases, and in addition, engage parallel routes in GTPase-independent manners [9]–[17]. The physiological role of Vav proteins in the immune, nervous, and cardiovascular systems are now well established thanks to the use of genetically engineered mice [9],[18]–[25]. By contrast, the genetic analysis of the role of these proteins in cancer has been restricted so far to acute lymphoblastic leukemia and polyomavirus middle T-antigen-induced breast cancer. These studies have revealed that Vav3 and Vav2 plus Vav3 were required for the development of each of those tumors, respectively [7],[17].

In the present work, we aimed at expanding the spectrum of Vav family-dependent tumors by focusing our attention on cutaneous squamous tumors (CSTs), the second most frequent type of skin cancer worldwide [26]. To this end, we decided to use *Vav* family knockout mice to evaluate the role of these proteins in the development of 7,12-dimethylben[*a*]antracene (DMBA)/12-O-tetradecanoylphorbol-13-acetate (TPA)- and DMBA/DMBA-triggered skin tumors. In the former model, a single topic administration of DMBA induces oncogenic mutations (Q61L) in the *HRas* locus (ID number: 15461) in a small pool of keratinocytes (initiation phase). Subsequent serial topic applications of TPA are then applied to expand this pool of transformed keratinocytes to generate papillomas (promotion phase) and, depending on the genetic background of mice, cutaneous squamous cell carcinomas (cSCCs) (progression phase). Such pro-tumorigenic effect is mediated by the stimulation of intracellular signaling cascades in the initiated keratinocytes and, in addition, through autocrine/paracrine-based crosstalk between cancer and tumor-associated stromal cells that ultimately favor the expansion of the initial pool of transformed keratinocytes [27]–[30]. The latter model uses serial topic applications of DMBA that increase the frequency of cSCC development at the end of the carcinogenic protocol. We selected these models for our experiments because: (i) they are known to be Rac1- (ID number: 19353) and Tiam1-dependent [4],[31]; (ii) high levels of two Vav family proteins and a large number of additional Rho GEFs are present in normal and tumoral skin (see Figure S1), thus making a perfect working model to address intra-GEF family redundancies in a tumorigenic context; and (iii) they are compatible with the analysis of the role of the proteins under study in the tumor initiation, promotion, and progression phases [28]. Using this strategy, we have unveiled a Vav2/Vav3-dependent and cancer-specific autocrine/paracrine program that contributes to the initiation and promotion phases of skin tumors.

## Results

### Vav2 and Vav3 Proteins Are Essential for Skin Tumorigenesis

**Figure 1:**
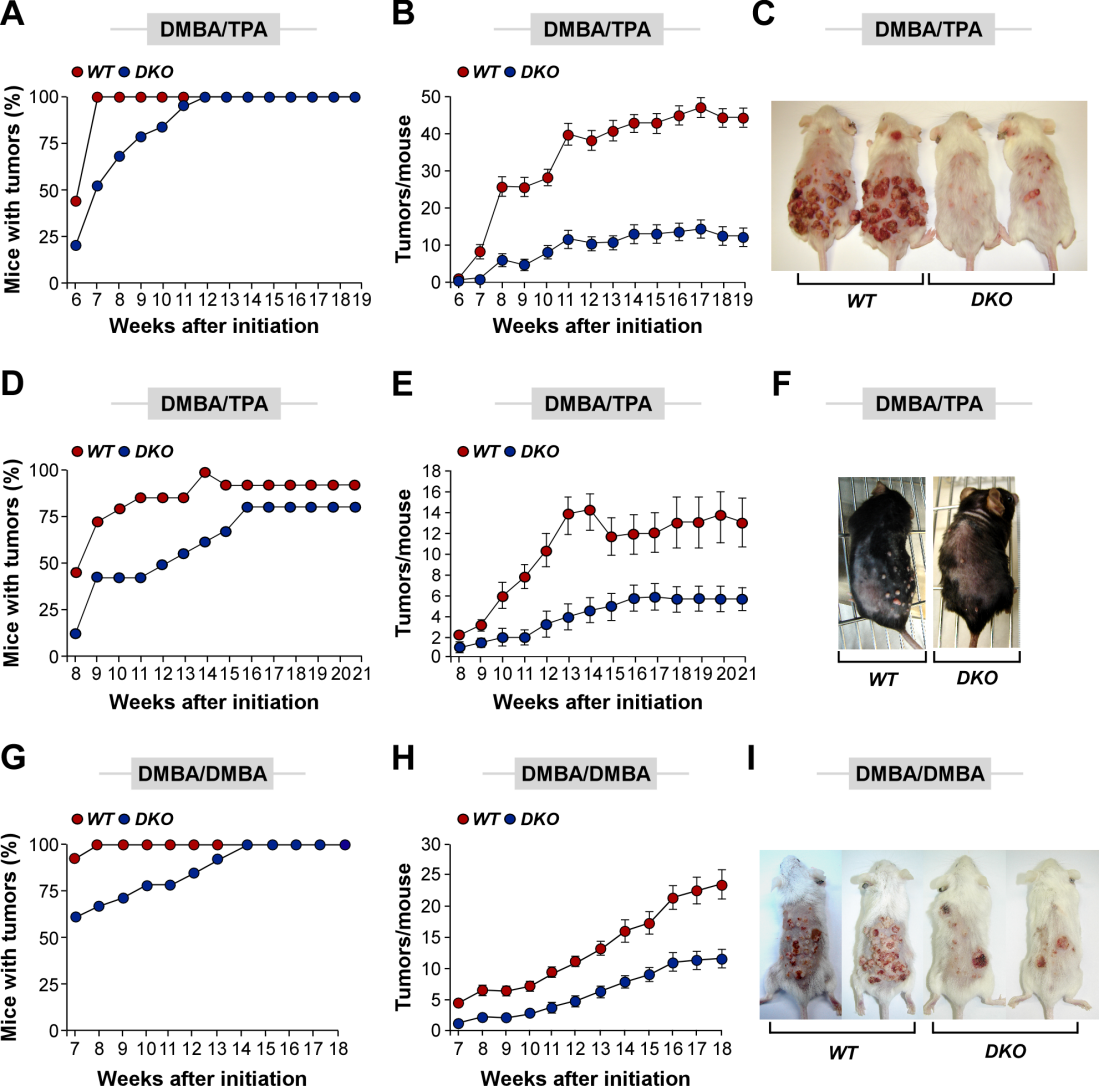
Expression of Vav2 and Vav3 is important for CST development. (A, B) Incidence (A) and number (B) of tumors in DMBA/TPA-treated FVB mice of indicated genotypes. The differences in tumor incidence up to week 10 (A) and in tumor number (B) were statistically significant (*p*≤0.001, *n* = 15 animals per genotype in each experiment). (C) Example of animals subjected to the above treatments for 16 wk. (D, E) Incidence (D) and number (E) of tumors in DMBA/TPA-treated C57BL/10 mice of indicated genotypes. The differences in tumor incidence up to week 15 (D) and in tumor number (E) were statistically significant (*p*≤0.001, *n* = 15 animals per genotype in each experiment). (F) Example of C57BL/10 mice of the indicated genotypes that were subjected to the above treatment for 16 wk. (G, H) Incidence (G) and number (H) of tumors in DMBA/DMBA-treated FVB mice of indicated genotypes. The differences in tumor incidence up to week 13 (G) and in tumor number (H) were statistically significant (*p*≤0.001, *n* = 15 animals per genotype in each experiment). (I) Example of animals subjected to the above treatments for 16 wk.

Expression analyses indicated that mouse papillomas and cSCCs expressed large numbers of Rho GEFs, including Vav2 and Vav3 (Figure S1). To assess if these two Vav family proteins played nonredundant roles with other Rho GEFs in the skin, we used compound *Vav2* ^−/−^;*Vav3* ^−/−^ mice to evaluate the impact of the systemic inactivation of these two proteins in both epidermal maintenance and tumorigenesis. Unlike the case of *Rac1* ^−/−^ mice [32]–[34], we could not find any defect in skin development, histological structure, or self-renewal in those mice regardless of the genetic background used (Figure S2 and unpublished data). Wild-type-like parameters were also found in triple *Vav1* ^−/−^;*Vav2* ^−/−^;*Vav3* ^−/−^ C57BL/10 mice, indicating that the lack of an epidermal phenotype was not due to functional compensation events by Vav1 (unpublished data). Hence, other Rho/Rac GEFs must be in charge of stimulating Rac1 in skin stem cells and keratinocytes under physiological conditions. By contrast, we observed that *Vav2* ^−/−^;*Vav3* ^−/−^ FVB mice displayed lower kinetics of tumor development (Figure 1A), a ≈5-fold reduction in the total number of tumors developed per mouse (Figure 1B,C), and 10-fold lower levels of carcinoma in situ (Table S1) when subjected to the two-step DMBA/TPA carcinogenic method. This effect was independent of the mouse genetic background, because C57Bl/10 *Vav2* ^−/−^;*Vav3* ^−/−^ mice also showed statistically significant reductions in tumor burden when compared to control animals (Figure 1D–F). *Vav2* ^−/−^;*Vav3* ^−/−^ FVB mice also displayed lower kinetics of tumor development (Figure 1G), a 2-fold reduction in tumor burden per mouse (Figure 1H,I), and a decrease in the percentage of cSCC development (Table S2) when subjected to the complete DMBA/DMBA tumorigenic method. Taken together, these results indicate that Vav2 and Vav3 play important roles in CST development but not in normal epithelial development and homeostasis.

### Vav Proteins Are Important for the Initiation Phase of Skin Tumorigenesis

**Figure 2:**
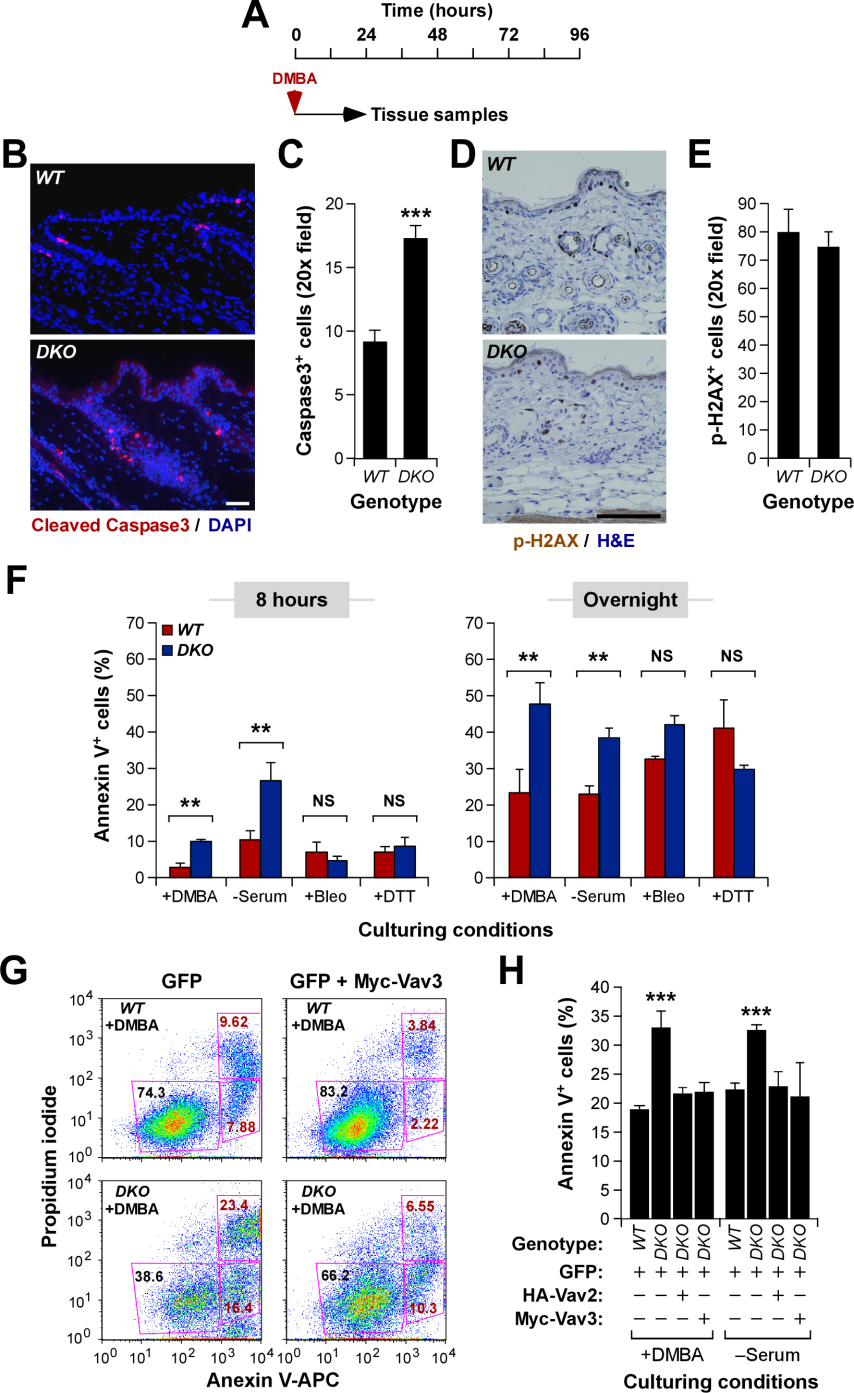
Vav proteins participate in the initiation phase of skin tumors. (A) Scheme of these experiments. (B, C) Example (B; scale bar, 100 µm) and quantification (C) of apoptotic cells found in the epidermis of mice of the indicated genotypes 24 h upon the application of DMBA (*n* = 4). In (B), sections were stained with antibodies to the cleaved fragment of caspase 3 (ID number: 12367) and 14, 4′,6-diamidino-2-phenylindole (DAPI) to reveal apoptotic cells (red color) and cell nuclei (blue color), respectively. (D, E) Example (D; scale bar, 100 µm) and quantification (E) of phospho-histone H2AX^+^ keratinocytes (brown color) present in the epidermis of short-term DMBA-treated mice of indicated genotypes (*n* = 4). p-, phosphorylated. (F) Number of apoptotic (annexin V^+^) wild-type and *Vav2* ^−/−^;*Vav3* ^−/−^ keratinocytes induced after 8 (left panel) and 12 (right panel) h in the indicated culture conditions (*n* = 4). Bleo, bleomycin; DTT, dithiothreitol; NS, no statistically significant. (G, H) Example of a flow cytometry experiment (G) and subsequent quantification (H) of the level of apoptosis induced by either DMBA (G, H) or serum starvation (H) in wild-type and *Vav2* ^−/−^;*Vav3* ^−/−^ keratinocytes ectopically expressing GFP either alone (G, H) or in combination with HA-Vav2 (H) or Myc-Vav3 (G, H) (*n* = 3). In (G), only the gated GFP^+^ cells are shown.

The lower tumor burden observed in DMBA/DMBA-treated *Vav2* ^−/−^;*Vav3* ^−/−^ mice indicated that Vav proteins may have direct roles during the initiation phase of CSTs. To investigate this possibility, we analyzed the short-term response of the epidermis of wild type and *Vav2* ^−/−^;*Vav3* ^−/−^ mice to DMBA (Figure 2A). Using immunostaining with antibodies to the cleaved fragment of caspase 3, we observed that DMBA induced ≈2-fold higher apoptotic cell numbers in the epidermis of *Vav2* ^−/−^;*Vav3* ^−/−^ mice than in controls (Figure 2B,C). This was not due to enhanced absorption and/or metabolization of the carcinogen, because immunohistochemistry experiments with antibodies to phospho-histone H2AX (ID number: 15270) indicated that the DMBA treatment triggered similar levels of DNA double-strand breaks in mice of both genotypes (Figure 2D,E). We also found that primary *Vav2^−/−^*;*Vav3* ^−/−^ keratinocytes were more susceptible to programmed cell death upon DMBA treatment or serum starvation in tissue culture, suggesting that the defects detected in vivo were keratinocyte autonomous (Figure 2F). This was a stimulus-dependent defect, because wild-type and mutant keratinocytes displayed similar cell death rates when challenged with other pro-apoptotic agents such as radiomimetic (bleomycin) and endoplasmic reticulum stress-inducing (dithiothreitol) drugs (Figure 2F). The ectopic expression of either HA-tagged Vav2 or Myc-tagged Vav3, but not of the control green fluorescent protein (GFP, ID number: P42212), restored wild-type-like apoptotic rates in both DMBA-treated and serum-starved *Vav2* ^−/−^;*Vav3* ^−/−^ keratinocytes (Figure 2G,H), further indicating that this survival defect was a direct effect of the *Vav2;Vav3* gene deficiency in keratinocytes.

### Vav Proteins Are Important for the Promotion Phase of Skin Tumorigenesis

**Figure 3:**
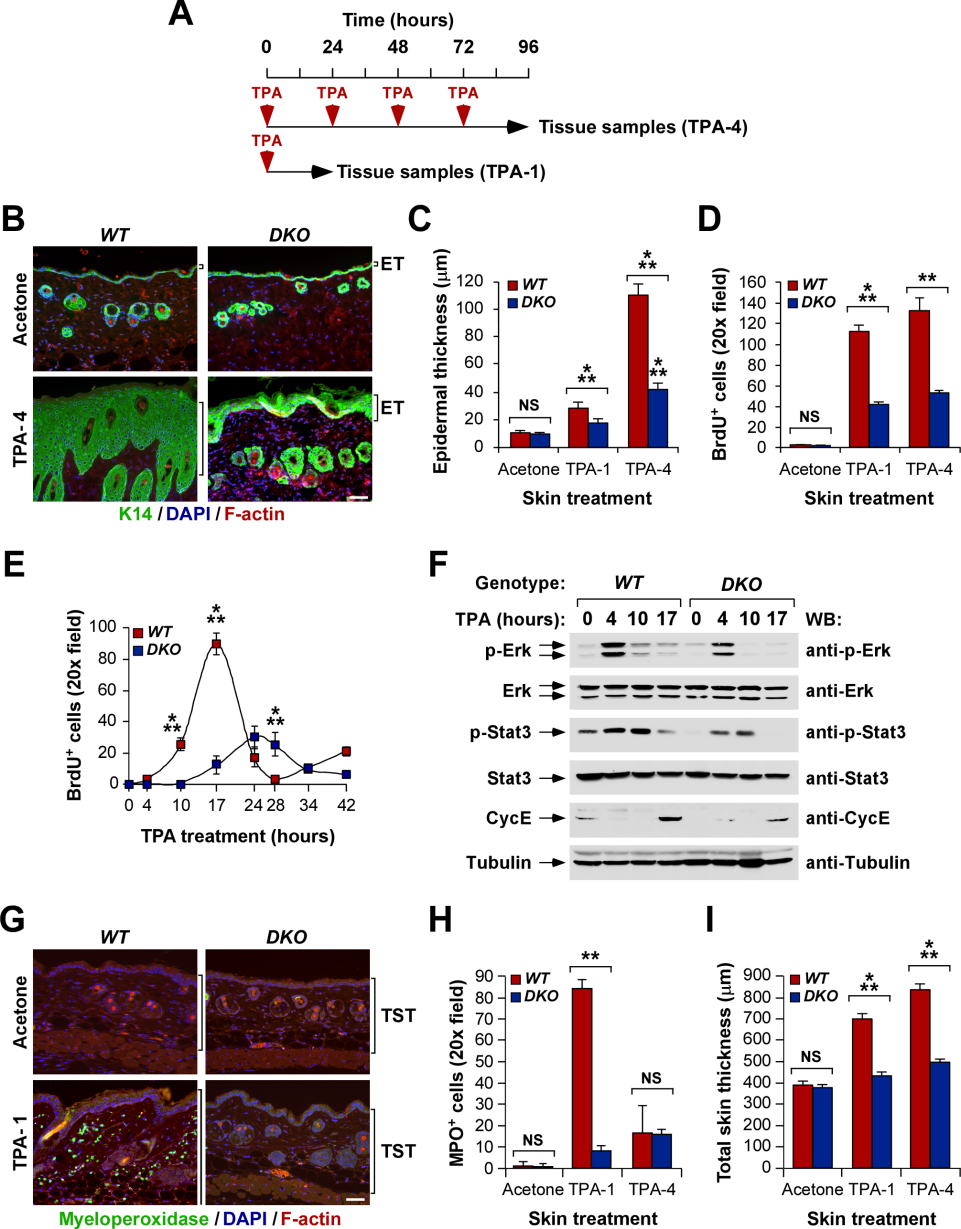
Vav proteins play pleiotropic roles during the skin tumor promotion phase. (A) Scheme of the experiments made in this section. (B–D) Example of immunofluorescence experiments (B; scale bar, 100 µm) and quantification of the hyperplasia (C, green color cells) and number of BrdU^+^ keratinocytes (D) in the epidermis of TPA-treated mice of indicated genotypes (*n* = 6). In (B), sections were stained with antibodies to keratin 14 (ID number: 16664), DAPI, and rhodamine-phalloidin to reveal K14 (green color), cell nuclei (blue color), and F-actin (red color), respectively. TPA-1 refers to tissue samples harvested either 24 (C) or 17 (D) h after the TPA stimulation. ET, epidermal thickness. (E) Quantification of the number of BrdU^+^ keratinocytes at the indicated times after a single topic application of TPA in animals of indicated genotypes (*n* = 3). (F) Phosphorylation and expression status of indicated proteins in the epidermis of wild-type and *Vav2* ^−/−^;*Vav3* ^−/−^ mice at the indicated times after a single topic application of TPA. Tubulin α (ID number: 22142) was used as loading control. WB, Western blot. (G–I) Example (G; scale bar, 100 µm) and quantification of the neutrophil infiltration (H, green color) and edema (I) induced by TPA in mice of indicated genotypes (*n* = 3). In (G), the sections were stained with antibodies to myeloperoxydase (MPO, ID number: 17523), DAPI, and rhodamine-phalloidin to decorate neutrophils (green color), nuclei (blue color), and F-actin (red color), respectively. TST, total skin thickness.

We hypothesized that Vav proteins could be also involved in the TPA-dependent promotion phase of skin tumors, an idea consistent with our prior observations indicating that the effect of the double *Vav2;Vav3* gene deficiency in the reduction of tumor burden was significantly more conspicuous in DMBA/TPA- than in DMBA/DMBA-treated mice (Figure 1; compare panels B and H). To investigate this possibility, we evaluated the short-term proliferative and inflammatory reaction induced by TPA in the skin of control and *Vav2* ^−/−^;*Vav3* ^−/−^ mice (Figure 3A). The TPA-induced proliferative response of the epidermis was severely attenuated in the absence of these two proteins, as demonstrated by the limited hyperplasia (Figure 3B,C) and the low levels of BrdU incorporation into keratinocytes (Figure 3D) detected in the epidermal layers of TPA-treated *Vav2* ^−/−^;*Vav3* ^−/−^ mice. In vivo BrdU pulse-chase experiments indicated that those proliferative defects were associated with delayed kinetics and a reduced efficiency in the G_1_/S phase transition induced by TPA in the mutant keratinocytes (Figure 3E). Consistent with this, immunoblot analyses using total cellular extracts obtained from the epidermis of TPA-treated mice showed that the activation (extracellular regulated kinase, [Erk, ID numbers: 26417, 26413], signal transduction and activator of transcription 3 [Stat3, ID number: 20848]) or abundance (cyclin E, ID number: 12447) of proteins involved in such cell cycle transition did not take place efficiently in *Vav2* ^−/−^;*Vav3* ^−/−^ mice (Figure 3F). Furthermore, we observed that the dermis of these animals did not show any sign of neutrophil infiltration (Figure 3G,H) or edema-associated thickening (Figure 3G,I), indicating that the inflammatory response that takes places during the tumor promotion phase is totally abated in the absence of Vav proteins.

**Figure 4:**
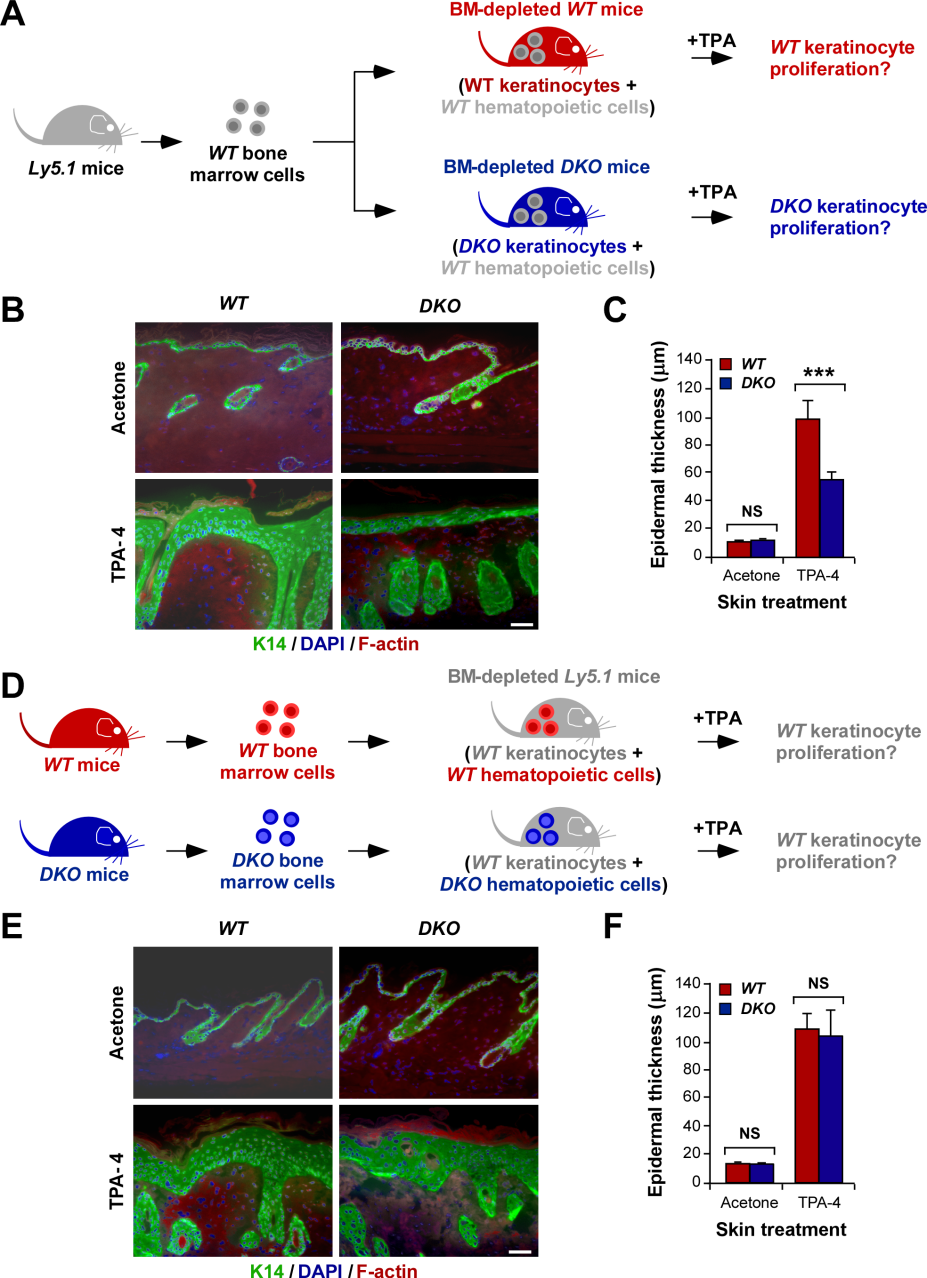
The defective proliferation of the epidermis of *Vav2* ^−/−^;*Vav3* ^−/−^ mice is not due to a defective hematopoietic system. (A) Scheme of the bone marrow reconstitution experiments used to generate data in panels (B) and (C). BM, bone marrow. (B, C) Example (B; scale bar, 100 µm) and quantification (C) of the epithelial hyperplasia induced upon four daily applications of TPA in the skin of wild-type and *Vav2* ^−/−^;*Vav3* ^−/−^ C57BL/10 mice previously reconstituted with a wild-type hematopoietic system (C57BL/6-Ly5.1 genetic background) (*n* = 3 animals/genotype). In (B), sections were stained with antibodies to K14, DAPI, and rhodamine-phalloidin to reveal K14 (green color), cell nuclei (blue color), and F-actin (red color), respectively. (D) Scheme of the bone marrow reconstitution experiments used to generate data in panels (E) and (F). (E, F) Example (E; scale bar, 100 µm) and quantification (F) of the epidermal thickness induced by four daily applications of TPA in the skin of wild-type C57BL/6-Ly5.1 mice whose hematopoietic systems had been reconstituted with either wild-type or *Vav2* ^−/−^;*Vav3* ^−/−^ bone marrow cells (C57BL/10 genetic background) (*n* = 3 animals/genotype). Panels shown in (E) were stained as in panel (B).

The lack of an inflammatory response led us to explore the potential contribution of *Vav2* ^−/−^;*Vav3* ^−/−^ inflammatory cells to the proliferative defects found in the epidermis of *Vav2* ^−/−^;*Vav3* ^−/−^ mice. We observed that such defects still persisted in *Vav2* ^−/−^;*Vav3* ^−/−^ C57BL/10 mice carrying a wild-type C57BL/6-Ly5.1 hematopoietic system (Figure 4A–C), ruling out the possibility that the defective proliferation of the epidermis of the knockout animals could be an indirect consequence of dysfunctional hematopoietic cells. In agreement with those results, we also found that the grafting of *Vav2* ^−/−^;*Vav3* ^−/−^ C57BL/10 bone marrow cells into lethally irradiated wild-type C57BL/6-Ly5.1 mice did not have any detectable effect on the responsiveness of the epidermis of host animals to TPA (Figure 4D–F).

### Vav Proteins Regulate a Mitogenic, Protein Kinase C/Fyn-Dependent Route in Keratinocytes

**Figure 5:**
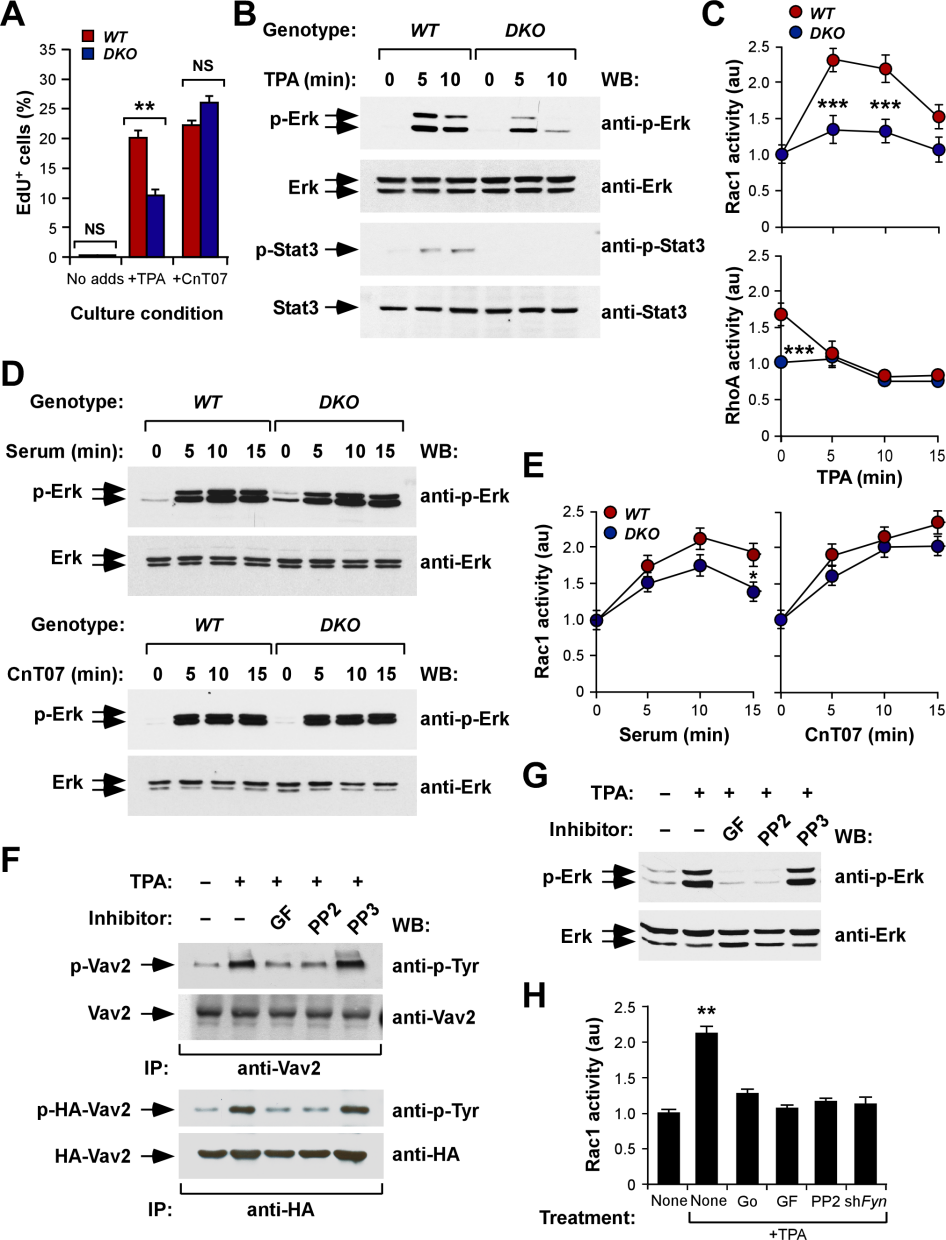
Vav-dependent routes in keratinocytes. (A) Quantification by flow cytometry of the number of EdU^+^, S-phase cells induced by the stimulation of quiescent keratinocytes of the indicated genotypes with either TPA or a synthetic keratinocyte growth media (CnT07). (B) Phosphorylation and expression status of Erk and Stat3 proteins in TPA-stimulated keratinocytes of indicated genotypes. (C) Rac1 (upper panel) and RhoA (lower panel) activation levels induced by the stimulation of quiescent keratinocytes of indicated genotypes with TPA (*n* = 3). (D) Phosphorylation and expression status of Erk proteins in either serum- (two upper panels) or CnT07-stimulated (two bottom panels) keratinocytes of indicated genotypes. (E) Rac1 activation levels induced by the stimulation of quiescent keratinocytes with either serum (left panel) or CnT07 media (right panel) (*n* = 3). (F) Immunoprecipitation experiments showing the tyrosine phosphorylation levels of endogenous Vav2 (top panel) and ectopically expressed HA-Vav2 (third panel from top) in wild-type keratinocytes treated with TPA (+) in the absence (−) or the presence (+) of the indicated drugs (*n* = 2). Due to problems with the detection of the endogenous proteins after blot stripping, the loading control shown in the second panel from top was made using a parallel immunoprecipitation with the same anti-Vav2 antibody. GF, GF109203X; IP, immunoprecipitation. (G) Western blot of total cellular extracts showing the levels of phosphorylation (top) and expression (bottom) of Erk proteins in wild-type keratinocytes stimulated as indicated in (F) (*n* = 3). (H) Rac1 activation levels induced by TPA in wild-type keratinocytes under the indicated experimental conditions (*n* = 3). Go, Gö 6976; sh*Fyn*, cells infected with a Fyn-specific shRNA.

To further assess the keratinocyte autonomous nature of the proliferative defects found in the epidermis of *Vav2* ^−/−^;*Vav3* ^−/−^ mice, we evaluated the proliferative response of wild-type and *Vav2;Vav3*-deficient primary keratinocytes to TPA in cell culture. In agreement with the in vivo data, we observed that quiescent *Vav2* ^−/−^;*Vav3* ^−/−^ keratinocytes incorporated less efficiently the S phase marker 5-ethynyl-2′-deoxyuridine (EdU) than their wild-type counterparts upon TPA stimulation (Figure 5A). This was a TPA-specific defect, because *Vav2* ^−/−^;*Vav3* ^−/−^ keratinocytes showed normal cell cycle progression when stimulated with complete growth media (Figure 5A). Western blot and GTPase-linked immunosorbent (G-LISA) assays revealed that the *Vav2;Vav3* gene deficiency was associated to reduced amounts of activation of Erk (Figure 5B, upper panel), Stat3 (Figure 5B, third panel from top), and Rac1 (Figure 5C, upper panel) in TPA-stimulated cells. It also reduced the basal levels of RhoA (ID number: 11848) activation in nonstimutated cells and, in addition, eliminated the inactivation of RhoA that was typically observed in TPA-stimulated wild-type keratinocytes (Figure 5C, lower panel). Normal levels of Erk and Rac1 activation were observed upon overexpression of HA-tagged Vav2 (Figure S3A,C) or Myc-tagged Vav3 in *Vav2;Vav3*-deficient keratinocytes (Figure S3B,C), thus confirming that those defects were directly due to the *Vav2;Vav3* gene deficiency. Furthermore, and consistent with the TPA-specific deficiency of the cell cycle transitions, we observed that those signaling responses were normal when *Vav2* ^−/−^;*Vav3* ^−/−^ keratinocytes were stimulated with either serum or synthetic CnT07 media (Figure 5D,E).

We surmised that a tyrosine kinase had to be involved in this process, because Vav proteins cannot be activated by direct TPA/diacylglycerol binding or protein kinase C (PKC)–mediated serine/threonine phosphorylation 9,35. In agreement with this idea, we observed that TPA triggered the tyrosine phosphorylation of both endogenous and ectopically expressed Vav2 in keratinocytes (Figure 5F). This phosphorylation was blocked when cells were pre-incubated with either general PKC (GF109203X) or Src family (PP2) inhibitors (Figure 5F) prior to the TPA stimulation step. An inactive PP2 analog (PP3) did not have such inhibitory effect on Vav2 tyrosine phosphorylation (Figure 5F). Similar results were obtained with the TPA-mediated stimulation of Erk route (Figure 5G). Although many PKC family members are present in keratinocytes (Figure S4A), we believe that a classical PKC (cPKC) must be involved, because we could reproduce the lack of Rac1 activation typically seen in *Vav2* ^−/−^;*Vav3* ^−/−^ keratinocytes when we incubated the wild-type counterparts with Gö6976 (Figure 5H), a cPKC-specific inhibitor that does not inactivate PKCs belonging to the novel or atypical subclasses [36]. A similar effect was observed when Fyn (ID number: 14360) was knocked down in wild-type cells using short hairpin RNA (shRNA) techniques (Figures 5H and S4B), indicating that this kinase was the Src family member preferentially involved in this signaling response. This is consistent with previous results reporting the TPA-mediated stimulation of this kinase in mouse keratinocytes [37]. These results indicate that Vav proteins act downstream of a cPKC/Fyn signaling route that mediates the pro-mitogenic effects of TPA in keratinocytes.

### Vav Proteins Promote a Large Transcriptomal Program During the Promotion Phase of Skin Tumors

We next considered the possibility that Vav proteins could control, in addition to the intrinsic signaling programs of keratinocytes described above, the stimulation of long-range autocrine/paracrine programs in the skin. This idea was consistent with the long-term defects seen in the activation/expression of G_1_/S phase-related signaling proteins in the epidermis of TPA-stimulated *Vav2;Vav3*-deficient mice (Figure 3F) and, in addition, by the total lack of inflammatory response found in the skin of those mice (Figure 3G–I). To explore this idea, we carried out microarray experiments to identify the fraction of the TPA-induced transcriptome of the skin (epidermis plus dermis) that was Vav-dependent. We found a significant subset of TPA-regulated transcripts whose upregulation (Figure S5A; right panel, A_1_ cluster; for functional annotation, see Table S3A,B) or repression (Figure S5A; right panel, A_2_ cluster; for functional annotation, see Table S3C,D) was Vav2/Vav3-dependent. Interestingly, bioinformatics analyses using a skin tumor microarray dataset [38] revealed that most A_1_ cluster transcripts displayed a similar up-regulation in DMBA/TPA-induced papillomas and/or cSCCs when compared to normal skin (Figure S5B, see clusters 2 and 3). Conversely, most A_2_ cluster mRNAs were expressed in normal skin and down-regulated in papillomas and/or cSCCs (Figure S5B, see clusters 5 and 6). These results indicated that the short-term Vav2/Vav3-dependent gene signature identified in the above microarray experiments is mostly conserved in fully developed tumors.

The functional annotation of the A_1_ gene cluster revealed a statistically significant enrichment of genes encoding extracellular ligands, including EGF family members (i.e., amphiregulin [Areg, ID number: 11839], tumor growth factor α [TGFα, ID number: 21802], heparin-binding EGF-like growth factor [HbEGF, ID number: 15200]), hepatocyte growth factor (HGF, ID number: 15234), fibroblast growth factor 7 (FGF7, ID number: 14178), vascular endothelial growth factor β (VEGFβ, ID number: 22340), and a large cohort of cytokines and chemokines (i.e., IL1β [ID number: 16176], interleukin 6 [IL6, ID number: 16193]) (Table S4). The detection of cytokine-encoding transcripts was not due to the infiltration of hematopoietic cells in the samples analyzed, because the A_1_ cluster did not include myeloid- or lymphocyte-specific genes (Table S3A). *In silico* analyses indicated that many of the genes for those ligands could be regulated by transcriptional factors belonging to the Stat (*p* = 6.4×10^−6^), nuclear factor of activated T-cells (NFAT; *p* = 0.002), nuclear factor kappa-light-chain-enhancer of activated B cells (NFκB; *p* = 0.004), AP1 (*p*≤0.05), and E2F (*p* = 0.03) families.

We confirmed by quantitative RT-PCR (qRT-PCR) that Vav proteins were important for the TPA-mediated induction of mRNAs for EGF family ligands, HGF, FGF7, IL6, and IL1β both in vivo (Figure S5C) and in vitro (Figure S5D). The only exception found for the correlation between whole skin and cultured keratinocytes was the *Tgfa* mRNA, which showed a Vav-dependent expression pattern in TPA-stimulated skin (Figure S5C) but not in isolated primary keratinocytes (Figure S5D). These results suggest that some of the transcripts detected in the skin microarray experiments probably represent secondary waves of transcriptional activation set in place upon the engagement of other Vav2/Vav3-dependent extracellular ligands. Defects in the production of HGF and IL6 by *Vav2;Vav3*-deficient mice were confirmed at the protein level using ELISA determinations in skin and serum samples obtained from TPA-treated animals (Figure S6A) and, in the case of IL6, by carrying out immunohistochemical analyses in skin sections (Figure S6B). The TPA-mediated up-regulation of these Vav-dependent transcripts was abolished in wild-type keratinocytes upon the *Fyn* mRNA knockdown (Figure S7), indicating that this autocrine/paracrine program is one of the downstream responses triggered by the TPA/cPKC/Fyn/Vav pro-mitogenic route previously characterized in keratinocytes (see above, Figure 5).

This Vav-dependent autocrine/paracrine program was keratinocyte-specific, since it was mostly absent in the recently described Vav-dependent transcriptome of mouse breast cancer cells [17]. Indeed, these two transcriptomes only shared the *Areg*, *Hbegf*, *Tnfa*, *Il24* (ID number: 93672), *Il23a* (ID number: 83430), *Osm* (ID number: 18413), and *Cxcl14* (ID number: 57266) transcripts (Table S4). By contrast, we observed that the majority of the Vav2/Vav3-dependent transcripts previously found to be involved in the lung-specific metastasis of breast cancer cells was also present in the Vav-dependent transcriptome of TPA-stimulated skin (*Inhba* [ID number: 16323], *Ptgs2* [ID number: 19225], *Tacstd2* [ID number: 56753]; Figure S8) [17]. The only exception was *Ilk* (integrin-linked kinase, ID number: 16202), which was repressed rather than induced by TPA independently of the expression status of Vav proteins (Figure S8A). This indicates that the Vav-dependent transcriptome contains both cell-type-dependent (i.e., the autocrine/paracrine program) and -independent (i.e., lung metastasis-related genes) subsets.

### The Vav Family-Dependent Autocrine/Paracrine Program Regulates Keratinocyte Survival and Proliferation

**Figure 6:**
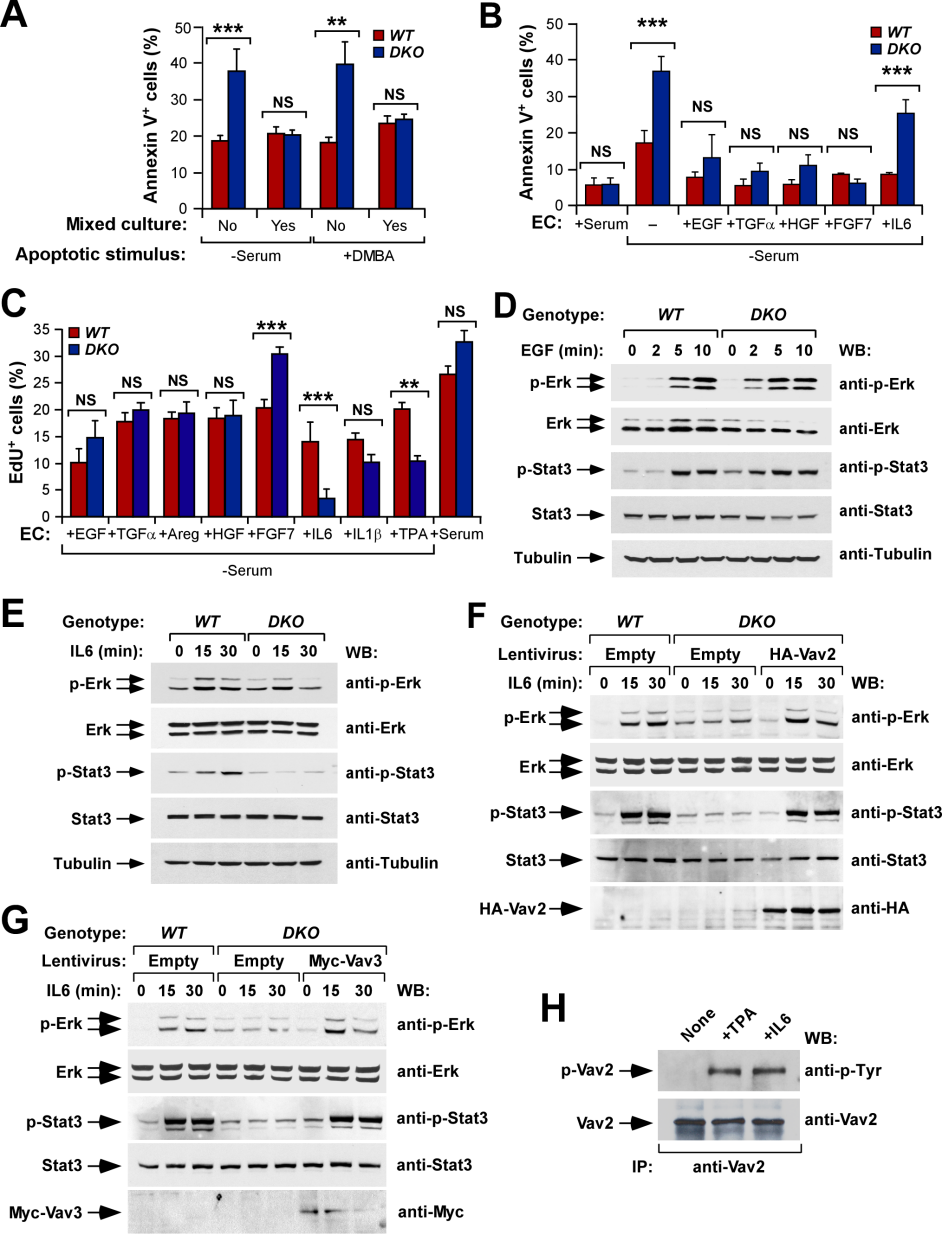
Vav proteins are involved in the engagement and signaling efficiency of keratinocyte autocrine/paracrine loops. (A) Quantification by flow cytometry of the apoptosis induced in pure (No) and genotypically mixed (Yes) cultures of wild-type and *Vav2* ^−/−^;*Vav3* ^−/−^ keratinocytes upon 12 h in complete media with DMBA or under serum-free media conditions. (B) Quantification by flow cytometry of the apoptosis observed in wild-type and *Vav2* ^−/−^;*Vav3* ^−/−^ keratinocytes kept for 12 h with serum, without serum, or without serum plus the indicated extracellular factors (*n* = 3). (C) Determination by flow cytometry of the percentage of wild-type and *Vav2* ^−/−^;*Vav3* ^−/−^ keratinocytes that have entered S phase after 4.5 h under the indicated culture conditions (*n* = 3). (D–F) Phosphorylation and expression status of Erk and Stat3 in serum-starved wild-type and *Vav2* ^−/−^;*Vav3* ^−/−^ keratinocytes stimulated with either EGF (D) or IL6 (E–G) for the indicated periods of time. In panels (F) and (G), keratinocytes were transduced with empty and Vav-encoding lentiviruses prior to the starvation and stimulation steps as indicated in the respective panel (*n* = 3). (H) Tyrosine phosphorylation levels of endogenous Vav2 (top panel) in wild-type keratinocytes treated with the indicated agents for 5 min (*n* = 2). Due to problems with the detection of the endogenous proteins after blot stripping, the loading control shown in the second panel from top was made using a parallel immunoprecipitation with the same anti-Vav2 antibody.

Given the critical roles that extracellular factors play in both the initiation and promotion phase of skin tumors [29],[30], we investigated whether they could be involved in the tumorigenic defects observed in *Vav2* ^−/−^;*Vav3* ^−/−^ keratinocytes. If so, we speculated that the experimental manipulation of the extracellular environment had to rescue wild-type-like responses in them. To this end, we first checked whether the survival defects exhibited by DMBA-treated keratinocytes could be overcome when co-culturing them in the presence of equal numbers of wild-type cells. To distinguish each cell subpopulation in the mixed cultures, one of the subpopulation was labeled with a cell permeable chromophore prior to the co-culturing step. Using annexin V flow cytometry, we found that wild-type cells restored normal survival rates to both DMBA and serum deprivation in the co-cultured *Vav2* ^−/−^;*Vav3* ^−/−^ keratinocytes (Figure 6A). A similar protective effect was found when we included, without wild-type cells, ligands for transmembrane tyrosine kinase receptors (epidermal growth factor [EGF, ID number: 13645], TGFα, HGF, FGF7) in the culture media of *Vav2* ^−/−^;*Vav3* ^−/−^ keratinocytes (Figure 6B). By contrast, the addition of IL6 protected wild type but not *Vav2* ^−/−^;*Vav3* ^−/−^ keratinocytes in the same type of experiments (Figure 6B).

The cell cycle defects shown by those cells under TPA-stimulation conditions were also eliminated when the serum-free media was supplemented with either transmembrane tyrosine kinase receptor ligands or IL1β (Figure 6C). However, as in the apoptotic assays, we observed that IL6 could induce cell cycle entry in wild-type but not in Vav-deficient keratinocytes (Figure 6C). This lack of responsiveness was not due to abnormal expression of any of the two IL6 receptor (IL6-R) subunits (Figure S9), indicating that *Vav2;Vav3*-deficient keratinocytes have, in addition to the general defect in the generation of the autocrine/paracrine program, a specific signaling defect downstream of the IL6-R. Consistent with this idea, immunoblot analyses indicated that EGF (Figure 6D), but not IL6 (Figure 6E), could trigger proper phosphorylation levels of Erk and Stat3 in *Vav2* ^−/−^;*Vav3* ^−/−^ keratinocytes. This was a direct consequence of the *Vav2;Vav3* gene deficiency, because we could restore normal phosphorylation levels of Erk and Stat3 downstream of the IL6-R upon the re-expression of either HA-tagged Vav2 (Figure 6F) or Myc-tagged Vav3 (Figure 6G) in *Vav2* ^−/−^;*Vav3* ^−/−^ cells. The implication of Vav proteins in the signaling of the IL6-R was further demonstrated by the observation that IL6 triggered tyrosine phosphorylation of endogenous Vav2 in wild-type keratinocytes (Figure 6H).

### The Vav Family-Dependent Autocrine/Paracrine Program Is Involved in the Promotion Phase of Skin Tumorigenesis

**Figure 7:**
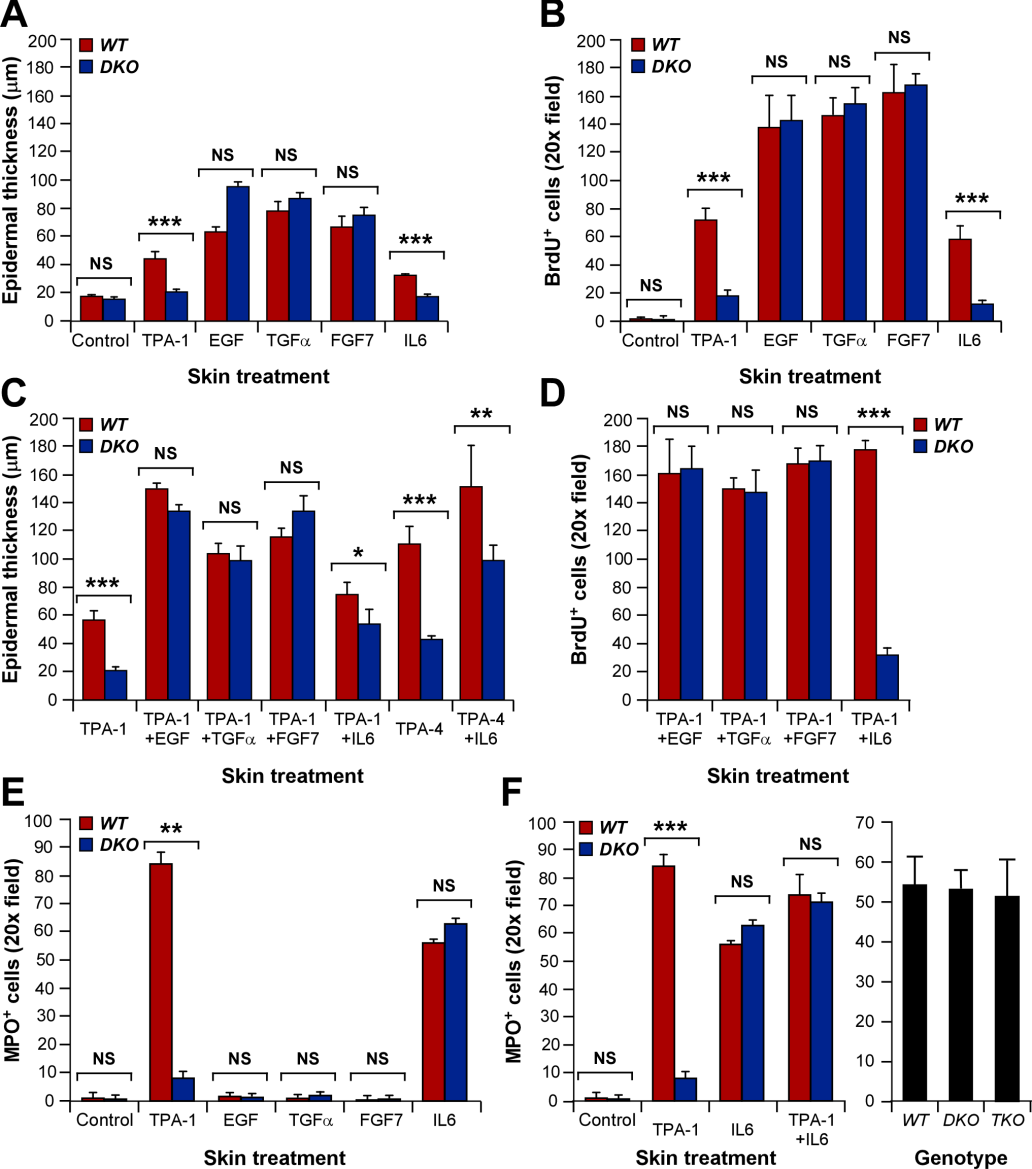
The defects of Vav2/Vav3-deficient mice in the promotion phase of skin tumorigenesis have an autocrine/paracrine basis. (A–D) Quantification of the epidermal hyperplasia (A, C) and BrdU incorporation (B, D) observed in wild-type and *Vav2* ^−/−^;*Vav3* ^−/−^ mice upon 24 (A, C) or 17 (B, D) h after indicated treatments (*n* = 4). TPA and extracellular factors were applied topically and intradermally, respectively. (E–F) Quantification of the neutrophil infiltration in wild-type (E, F), *Vav2* ^−/−^;*Vav3* ^−/−^ (E, F), and *Vav1* ^−/−^;*Vav2* ^−/−^;*Vav3* ^−/−^ (*TKO*) (F) mice upon 24 h treatments with the indicated stimuli (*n* = 4). In panel (F), the right graph represents data obtained upon IL6 injections. TPA and extracellular factors were applied topically and intradermally, respectively.

To further evaluate the relevance of the Vav-dependent autocrine/paracrine program, we investigated whether the intradermal injection of mitogens could eliminate the proliferative and inflammatory defects seen in the skin of TPA-treated *Vav2* ^−/−^;*Vav3* ^−/−^ mice. We observed that the injection of EGF, TGFα, or FGF7 induced similar levels of hyperplasia (Figure 7A) and BrdU immunoreactivity (Figure 7B) in the epidermis of wild-type and *Vav2;Vav3*-deficient mice. The simultaneous application of TPA resulted in a synergistic proliferative response in the epidermis of animals of both genotypes (Figure 7C,D). The intradermal injection of IL6 could trigger a robust, TPA-like mitogenic response in the epidermis of control but not *Vav2* ^−/−^;*Vav3* ^−/−^ animals (Figure 7A–D), thus recapitulating the signaling defects observed in primary keratinocyte cultures (see above; Figure 6). IL6, but not the other ligands tested, did induce a potent infiltration of neutrophils in the skin of both wild-type and knockout mice (Figure 7E,F). This indicates that, unlike the case of keratinocytes, the IL6-R does not require the presence of Vav2 and Vav3 for proper signaling in myeloid cells. This is not due to functional compensation events from Vav1, because IL6-treated *Vav1* ^−/−^;*Vav2* ^−/−^;*Vav3* ^−/−^ mice showed neutrophil infiltration rates similar to both wild-type and *Vav2* ^−/−^;*Vav3* ^−/−^ animals (Figure 7F, right panel).

### Carcinomas from *Vav* Knockout Mice Show Exacerbated Production of Mitogens

**Figure 8:**
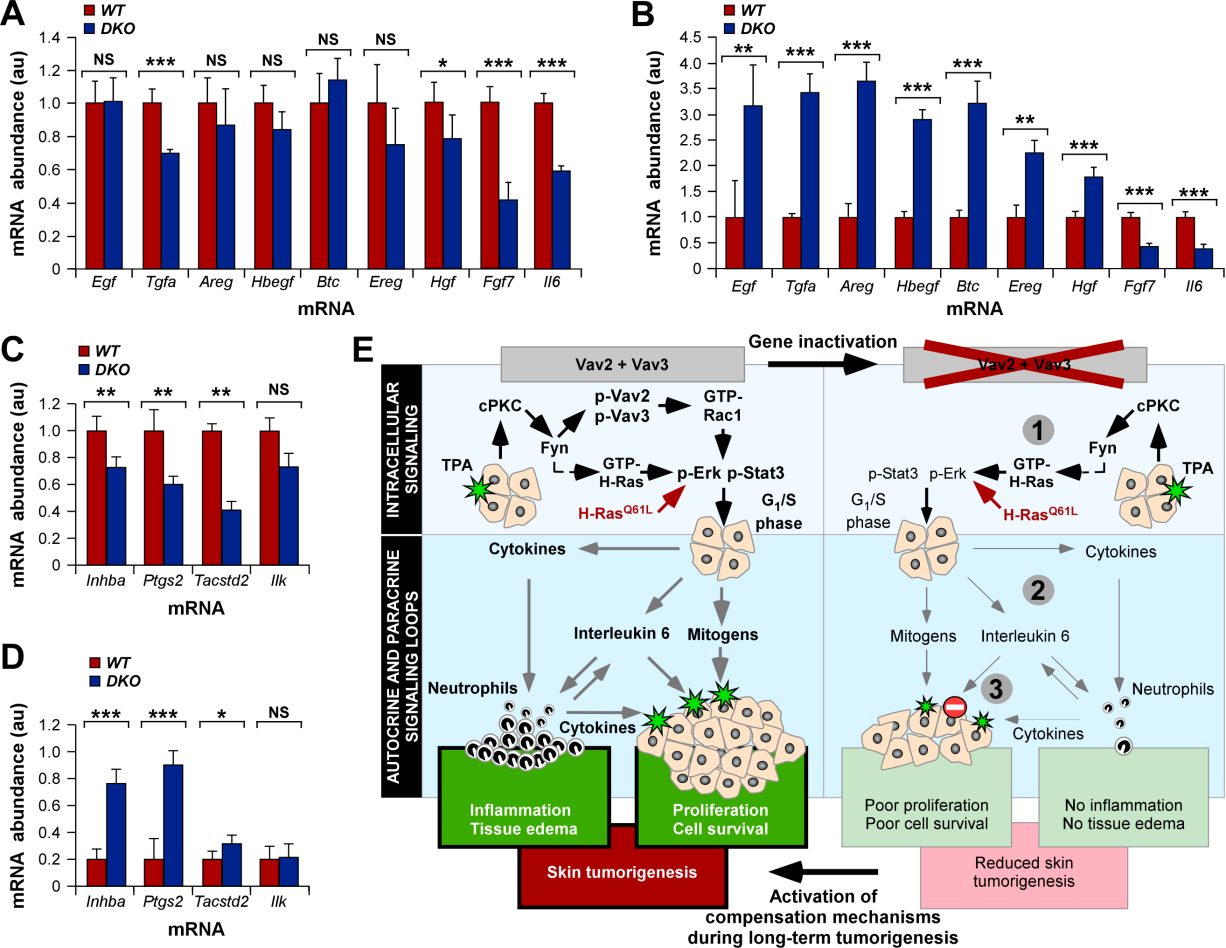
Progressive upr-egulation of EGF family ligands and Vav family-dependent genes in skin tumors developed in *Vav2* ^−/−^;*Vav3* ^−/−^ mice. (A–D) qRT-PCR experiments showing the relative abundance of indicated mRNAs in DMBA/TPA-induced papillomas (A, C) and DMBA/DMBA-induced carcinomas (B, D) in wild-type and *Vav2* ^−/−^;*Vav3* ^−/−^ FVB mice. Values for each mRNA are given in relation to the levels seen in samples from control mice (which were given an arbitrary value of 1) (*n* = 4 mice per genotype). (E) Summary of the results obtained in this work. (Left panel) The Vav2/Vav3-dependent biological programs in the skin that favor tumor initiation and promotion. (Right panel) The above programs upon the deletion of *Vav2* and *Vav3* genes. Numbered gray circles indicate the three main signaling functions of Vav proteins. The intracellular and extracellular signaling programs are indicated on the left boxes. Intracellular and autocrine/paracrine signaling is indicated by black and gray arrows, respectively. The oncogenic route is shown in red. Stimulation steps are shown as green stars. Defective receptor signaling is induced as a stop sign. Normal and defective processes are shown in bold and normal font types, respectively.

Finally, we investigated whether the tumors that developed in *Vav2;Vav3*-deficient mice could be the result of the outgrowth of cancer cells that, due to selection events, could have compensated the lack of Vav proteins by the exacerbation of other signaling routes. We suspected that such compensation could occur in this case because the few tumors that developed in mutant mice displayed a high similarity to those found in control animals in terms of size distribution, proliferation rates, and differentiation stage (unpublished data). Based on the above, we decided to compare the abundance of transcripts encoding a variety of mitogenic ligands in papilloma and cSCC samples obtained from FVB mice of both genotypes. In addition, we monitored the expression pattern in those samples of Vav2/Vav3-dependent genes that were common between breast cancer cells and TPA-stimulated keratinocytes [17]. We observed that the Vav2/Vav3-dependent *Tgfa*, *Hgf*, *Fgf7*, and *Il6* transcripts showed reduced abundance in papillomas derived from Vav2/Vav3-deficient mice when compared to the levels present in control mouse tumors (Figure 8A). This reduction, however, was milder than the defect originally seen in the TPA-stimulated skin of *Vav2* ^−/−^;*Vav3* ^−/−^ mice (see above, Figure S5C). Other Vav2/Vav3-dependent (*Areg*, *Hbegf*) and -independent (*Egf*, *Btc* [ID number: 12223]) transcripts displayed similar levels in papillomas regardless of the Vav2/Vav3 expression status, further suggesting that the autocrine/paracrine defect was ameliorated in these tumors (Figure 8A). Such compensation event was exacerbated in carcinomas, since we observed a striking up-regulation of the abundance of EGF-R family ligand transcripts in all cSCC samples derived from mutant mice (Figure 8B). Such up-regulation took place irrespectively of whether the ligands were of the Vav-dependent (*Tgfa*, *Areg*, *Hbegf*) or Vav-independent (*Egf*, *Btc*) subclasses (Figure 8B). Carcinomas from *Vav2;Vav3*-deficient mice also displayed up-regulation of the *Hgf* mRNA (Figure 8B). This was not a sign of a total elimination of the Vav2/Vav3 signaling deficiency, because lower levels of *Fgf7* and *Il6* transcripts were still detected in cSCCs collected from *Vav2* ^−/−^;*Vav3* ^−/−^ mice (Figure 8B). A similar behavior was observed in the case of the *Inhba* and *Ptgs2* when their abundance was compared between papilloma and cSCCs (Figure 8C,D). However, the *Tacstd2* and *Ilk* transcripts showed no variations in these experiments (Figure 8C,D). These results suggest that the HRas^Q61L^-transformed keratinocytes have to progressively bypass part of the Vav2/Vav3 signaling deficiency to generate fully developed tumors. This compensation event is not due to Vav1 overexpression, because this transcript shows a similar 3-fold increase in abundance in cSCCs of both wild-type and *Vav2* ^−/−^;*Vav3* ^−/−^ mice (*n* = 4).

## Discussion

Our work indicates that Vav2 and Vav3 play first- and second-level signaling roles in keratinocytes that, although mechanistically different, act in a concerted manner to favor the initiation and promotion phases of CSTs (Figure 8E). From a signaling hierarchical point of view, the earliest role of Vav proteins is to work in a cPKC- and Fyn-dependent signaling cascade that leads to the downstream stimulation of Rac1 and optimal phosphorylation kinetics of both Erk and Stat3 (Figure 8E, step 1). This route has a direct impact on the G_1_/S transition of primary keratinocytes. A more distal endpoint of this Vav-dependent route is the engagement of a wide autocrine/paracrine program that favors keratinocyte survival to DNA damage, epithelial hyperplasia, and the formation of an inflammatory microenvironment (Figure 8E, step 2). This program is composed of extracellular factors (i.e., HGF, FGF7, IL1β) involved in the monovalent regulation of keratinocyte survival and proliferation during the initiation and promotion phases, respectively. In addition, it contains bivalent extracellular factors (i.e., IL6) that regulate keratinocyte mitogenesis and the engagement of inflammatory responses during the tumor promotion phase. Finally, Vav proteins play a second-level, keratinocyte-specific role in the signaling route of one of the Vav2/Vav3-dependent extracellular factors, the cytokine IL6 (Figure 8E, step 3). This biological program may play roles in both inchoate and fully established tumors, as indicated by the similar regulation of the TPA-induced Vav2/Vav3-dependent gene signature in papillomas and cSCCs obtained from DMBA/TPA-treated mice. By contrast, we have observed that the compound *Vav2;Vav3* gene deficiency does not induce any overt dysfunction in normal skin development and homeostasis, indicating that the Vav2/Vav3-dependent route only becomes functionally relevant in the epidermis under conditions that require increased signaling thresholds for the assembly of new, pathophysiological-specific programs. To our knowledge, this is the first demonstration of the implication of Vav proteins, Rho GEFs, or any Rho GTPase in the assembly of such large autocrine/paracrine program in either a physiological or pro-tumorigenic scenario.

Several indications support the idea that the upstream and downstream roles of Vav proteins in this autocrine/paracrine program are critical for the initiation and promotion phase of inchoate skin tumors and, probably, for long-term tumor sustenance. Thus, previous reports have shown that many of the Vav2/Vav3-dependent extracellular factors regulated by Vav2 and Vav3 contribute to keratinocyte survival, proliferation, and tumorigenesis [29],[30]. Furthermore, we have shown that all the defects detected in cultured *Vav2* ^−/−^;*Vav3* ^−/−^ keratinocytes can be effectively bypassed upon their co-culture with wild-type keratinocytes or, alternatively, upon the addition of specific Vav2/Vav3-dependent ligands (EGF family ligands, HGF, FGF7, IL1β) to their cultures. Similar results were observed when these rescue experiments were carried out in vivo using intradermal injections of those extracellular factors in *Vav2* ^−/−^;*Vav3* ^−/−^ mice. However, and in good agreement with the critical role of Vav proteins downstream of the IL6-R, IL6 could not restore the survival and mitogenic defects of Vav2/Vav3-deficient keratinocytes when used in vitro or in vivo. It is likely that these second-level signaling defects also contribute to the lower tumor burden observed in *Vav2* ^−/−^;*Vav3* ^−/−^ animals, since previous reports have shown that IL6-mediated signaling is essential for skin tumorigenesis [39]. Interestingly, we observed that IL6 did restore the defective inflammatory response observed in the skin of TPA-stimulated *Vav2* ^−/−^;*Vav3* ^−/−^ animals. This result indicates that IL6 is indeed part of the Vav2/Vav3-dependent pro-mitogenic and pro-inflammatory program of keratinocytes and, in addition, that the critical role of Vav proteins downstream of the IL6-R is keratinocyte-specific. The use of *Vav1* ^−/−^;*Vav2* ^−/−^;*Vav3* ^−/−^ mice has ruled out the possibility that the normal chemoattraction of *Vav2* ^−/−^;Vav3^−/−^ neutrophils induced by IL6 could be due to functional compensation events mediated by the hematopoietic-specific Vav1 protein [9]. Thus, these cell-type-specific differences could be due to the presence of other IL6-regulated Rho GEFs in neutrophils (e.g., P-Rex1) [40] or, alternatively, reflect the implication of Vav proteins in a proliferative/survival signaling branch of the IL6-R that is not important for migration and the induction of the pro-inflammatory program. Additional experiments using wild-type and Vav family-deficient neutrophils will be needed to discriminate those possibilities. Finally, the pathophysiological importance of this program during skin tumorigenesis is further highlighted by our in silico analyses indicating that the Vav2/Vav3-dependent transcriptomal signature is conserved in fully developed tumors and, in addition, by the exacerbated up-regulation of most transcripts for those autocrine/paracrine factors consistently seen in cSCCs from *Vav2* ^−/−^;*Vav3* ^−/−^ mice (Figure 8E).

We surmise that these experiments have only revealed the tip of the iceberg of this biological program, because the Vav2/Vav3-dependent transcriptomal signature encodes other pro-mitogenic and pro-inflammatory factors that have not been yet analyzed. It also contains factors with assigned roles in angiogenesis (i.e., Cyr61 [ID number: 16007], IL6, VEGFβ), the polarization of the T_H_ cell response towards the T_H1_ (i.e., Ccl3 [ID number: 20302], Ccl4 [ID number: 20303], Cxcl5 [ID number: 20311], IL1β, Spp1 [ID number: 20750], TNFα), and T_H17_ (i.e., Csf3 [ID number: 12985], Cxcl2 [ID number: 20310], Cxcl5, IL6, IL10 [ID number: 16153]) subtypes or the tumor-induced “education” of dermal fibroblasts (i.e., IL1β) [29],[30],[41]–[43], thus suggesting that the engagement of the Vav2/Vav3 route in keratinocytes could result in a widespread reprogramming of the tissue microenvironment (Figure 8E). This biological program is also endowed with multiple positive feedback mechanisms that can lead to self-amplification and long-term signal sustenance upon its initial engagement in keratinocytes. An obvious signaling relay is the Vav→IL6→IL6-R→Vav→Stat3 route, since it is known that the stimulation of phospho-Stat3 re-feeds this autocrine loop by activating transcriptionally the *Il6* gene [44],[45]. The targeted inflammatory and stromal cells provide additional options for amplification and diversification of signals, since those cells can turn on additional paracrine mechanisms that target keratinocytes and stromal cells [41]–[43]. Thus, it is likely that the initial activation of the Vav route in keratinocytes will kindle a “butterfly effect” that will induce widespread and reciprocal signaling interactions among keratinocytes, resident stromal cells, and newcomer inflammatory and immune cells.

Our work also sheds light on issues related to functional redundancies with the Rho GEF family and Vav subfamily during the initiation and promotion phases of skin tumors. On the one hand, the cell reconstitution experiments indicate that Vav2 and Vav3 seem to act redundantly in all the biological processes analyzed in this work. Consistent with this idea, we have observed that single *Vav2* ^−/−^ and *Vav3* ^−/−^ knockout mice do not show the initiation and promotion defects found in the compound *Vav2;Vav3*-deficient mice (M.M.-M. and X.R.B., unpublished data). On the other hand, this program seems to be quite idiosyncratic for Vav proteins as inferred by the detection of a phenotype despite the large number of Rho GEFs that, according to our bioinformatic array analyses, are present in normal skin, papilloma, and cSCCs. The cancer-linked phenotype of Vav2/Vav3-deficient mice is also quite different from that previously reported for Tiam1-deficient mice during both tumor initiation [4],[5] and papilloma/cSCC malignant progression [4]. In this context, the observation that subsets of Rho GEFs are differentially regulated in normal skin, papilloma, and cSCCs suggests the specific engagement of physiological- and tumor-stage-specific Rac1-, RhoA, and Cdc42 (ID number: 12540)-dependent programs that may contribute to both skin homeostasis and pathophysiology. These results emphasize the importance of extending animal-model-based genetic analysis to all Rho GEF family members.

Our results suggest that the pharmacological targeting of Vav proteins could be a potentially useful strategy in skin cancer. Since our data have been generated using mice lacking Vav proteins from the initiation stage, they could only be formally used to establish the value of such therapies at the prevention rather than the remediation level. However, preventive therapies are interesting in this case because CSTs are known to develop at high frequency and multiplicity in individuals with actinic keratosis or in patients treated with some immunosuppressants, antifungal antibiotics, or antitumoral therapies (i.e., B-Raf (ID number: 109880) inhibitors) [46]. Assessing the value of such potential therapies in the case of fully developed or metastasized CSTs will require the generation of chemically inducible knock-in systems. In any case, we can anticipate from the present data that anti-Vav therapies will elicit much fewer intrinsic side effects in the skin than those based on the inactivation of either Tiam1 or Rac1.

## Materials and Methods

### Ethics Statement

All animal work has been done in accordance with protocols approved by the Bioethics committees of both the University of Salamanca and CSIC.

### Expression of Rho GEFs Using Bioinformatic Analyses

To analyze the expression of Rho GEFs mRNAs in skin and tumors, raw data containing samples from normal tail skin (*n* = 83), papillomas (*n* = 60), and carcinomas (*n* = 68) was downloaded from the Gene Expression Omnibus website (Accession number: GSE21264). These data were obtained in Balmain's laboratory using Affymetrix Mouse Genome 430 2.0 arrays and animals belonging to a mixed *Mus musculus* FVB/*Mus spretus* genetic background [38]. Signal intensity values were obtained from CEL files after RMA. Probesets corresponding to Rho/Rac GEFs were extracted from the dataset and ANOVA analysis used to identify Rho/Rac GEF-encoding transcripts that were differentially expressed between normal tail, papillomas, and carcinomas (*p* value <0.01). For those genes with more than one probeset significantly deregulated, we selected the one with the lowest *p* value for graphic representation.

### Analysis of mRNA Abundance

Total RNA was extracted from the indicated cells using Trizol (Sigma) and quantitative RT-PCR performed using the QuantiTect SYBR Green RT-PCR kit (Qiagen) and the iCycler machine (Bio-Rad) or, alternatively, the Script One-Step RT-PCR kit (BioRad) and the StepOnePlus Real-Time PCR System (Applied BioSystems). Raw data were then analyzed using either the iCycler iQ Optical System software (Bio-Rad) or the StepOne software v2.1 (Applied Biosystems). We used the abundance of the endogenous *Gapdh* mRNA as internal normalization control. Primers used for transcript quantitation included 5′-TTG CCC AGA ACA AAG GAA TC-3′ (forward for mouse *Vav1*), 5′-AAG CGC ATT AGG TCC TCG TA-3′ (reverse for mouse *Vav1*), 5′-AAG CCT GTG TTG ACC TTC CAG-3′ (forward for mouse *Vav2*), 5′-GTG TAA TCG ATC TCC CGG GAT-3′ (reverse for mouse *Vav2*), 5′-GGG TAA TAG AAC AGG CAC AGC-3′ (forward for mouse *Vav3*), 5′-GCC ATT TAC TTC ACC TCT CCA C-3′ (reverse for mouse *Vav3*), 5′-GGC AAA AAG TCA GTC CGA CC-3′ (forward for mouse *Fyn*, transcript variants 1 and 2), 5′-AAA GCG CCA CAA ACA GTG TC-3′ (reverse for mouse *Fyn*, transcript variants 1 and 2), 5′-TCG TGG CAA AAG AGC TTG GA-3′ (forward for mouse *Fyn*, transcript variant 3), 5′-TAG GGT CCC AGT GTG AGA GG-3′ (reverse for mouse *Fyn*, transcript variant 3), 5′-CGT CCG CCA TCT TGG TAG AGA GAG CAT-3′ (forward for mouse *Cd3e*), 5′-CTA CTG CTG TCA GGT CCA CCT CCA C-3′ (reverse for mouse *Cd3e*), 5′-ATG CTA GCG ATG CAT GAG TG-3′ (forward for mouse *Tgfa*), 5′-CAG GGA CTT TCT TGC CTG AG-3′ (reverse for mouse *Tgfa*), 5′-CGG TGG AAC CAA TGA GAA CT-3′ (forward for mouse *Areg*), 5′-TTT CGC TTA TGG TGG AAA CC-3′ (reverse for mouse *Areg*), 5′-GCT GCC GTC GGT GAT GCT GAA GC-3′ (forward for mouse *Hbegf*), 5′-GAT GAC AAG AAG ACA GAC G-3′ (reverse for mouse *Hbegf*), 5′-GCA GAC ACC ACA CCG GCA CAA-3′ (forward for mouse *Hgf*), 5′-GCA CCA TGG CCT CGG CTT GC-3′ (reverse for mouse *Hgf*), 5′-TTT GGA AAG AGC GAC GAC TT-3′ (forward for mouse *Fgf7*), 5′-GGC AGG ATC CGT GTC AGT AT-3′ (reverse for mouse *Fgf7*), 5′-CTT CCT ACC CCA ATT TCC AAT G-3′ (forward for mouse *Il6*), 5′-ATT GGA TGG TCT TGG TCC TTA GC-3′ (reverse for mouse *Il6*), 5′-ACG GAC CCC AAA AGA TGA AGG GCT-3′ (forward for mouse *Il1b*), 5′-GGG AAC GTC ACA CAC CAG CAG G-3′ (reverse for mouse *Il1b*), 5′-CGC TGC TTT GTC TAG GTT CC-3′ (forward for mouse *Ereg*), 5′-GGG ATC GTC TTC CAT CTG AA-3′ (reverse for mouse *Ereg*), 5′-CCC AGG CAA CGT ATC AAA GT-3′ (forward for mouse *Egf*), 5′-CCC AGG AAA GCA ATC ACA TT-3′ (reverse for mouse *Egf*), 5′-GGA ACC TGA GGA CTC ATC CA-3′ (forward for mouse *Btc*), 5′-TCT AGG GGT GGT ACC TGT G-3′ (reverse for mouse *Btc*), 5′-TGC ACC ACC AAC TGC TTA GC-3′ (forward for mouse *Gapdh*), and 5′-TCT TCT GGG TGG CAG TGA TG-3′ (reverse for mouse *Gapdh*). Primers used for quantitating *Inhba*, *Ptgs2*, *Tacstd2*, and *Ilk* transcripts were described before [17]. To calculate the number of copies of mouse *Vav* family mRNAs in cell/tissue samples, the Ct values obtained for the amplified *Vav1*, *Vav2*, and *Vav3* cDNA fragments by qRT-PCR in each sample were compared with those obtained using serial dilutions of plasmids of known concentration containing the *Vav1* (pJLZ52) [13], *Vav2* (pCCM33) [17], and Vav3 (pCCM31) [17] cDNAs. The number of copies obtained for each transcript was finally calculated using this titration curve and the size of each plasmid.

### Animal Studies

Single and compound *Vav* family knockout mice were described elsewhere [19],[23],[47]–[49]. For long-term carcinogenesis experiments, the backs of animals of the indicated genotypes were shaved and, 2 d later, the two-step carcinogenic DMBA/TPA protocol initiated using a single topic application of DMBA (25 µg diluted in 200 µl of acetone, both from Sigma). The promotion phase consisted of biweekly applications of TPA (200 µl of a 1×10^−4^ M solution in acetone, Sigma) during a 20-wk-long period. For complete carcinogenesis, mice were treated biweekly with 5 µg of DMBA alone in 200 µl of acetone during 20 wk. The number, size (measured with a digital caliper), and incidence of papilloma was determined weekly. At the end of the experiment, animals were injected intraperitoneally with BrdU (100 µg/g body weight, Sigma) and euthanized 1 h later.

For short-term studies of in vivo epidermal apoptosis, the dorsal skin of mice was treated with a single application of either DMBA (25 nmol in 200 µl of acetone) or acetone (200 µl) 2 d after shaving. For short-term in vivo proliferation assays, the dorsal skin of mice was treated with either one or four applications of either TPA (6.8 nmol in 200 µl acetone) or carrier solution 2 d after shaving. Animals were injected with BrdU and euthanized, as indicated above. For the determination of in vivo cell cycle transitions, animals were treated with a single topic application of TPA and, 1 h before euthanasia, injected intraperitoneally with BrdU. Alternatively, TPA-treated animals were euthanized and the epithelial layer obtained to generate tissue cell extracts.

For intradermal injection of mitogens and cytokines, mice were anesthetized and injected under the epidermal layer of their skin with 10 µl of either the appropriate protein-containing saline solution or the control buffer injected using a 30-gauge needle and a Hamilton microsyringe. The concentration of ligands (Peprotech) in the injection solution was 100 ng/ml. Mice were then BrdU labeled and killed as above.

For bone marrow reconstitution experiments, 3–5×10^6^ cells of donor bone marrow cells were injected intravenously into recipient mice that were previously subjected to sublethal doses of irradiation (600 rad; 1 Gy = 100 rads). In these experiments, we used “wild-type” C57BL/6 Ly5.1 mice in order to distinguish the hematopoietic populations derived from them (which express the CD45.1 surface marker) and from the knockout C57BL/10 mice (expressing the CD45.2 surface marker). Reconstitution experiments were done in both orientations. Eight weeks after injection, peripheral blood samples were collected from the cheek vein and the proper reconstitution of the immune system by the injected donor cells evaluated using flow cytometry. Surface markers used in the cytometry experiments included an allophycocyanin-labeled mouse monoclonal antibody to CD45.2, a biotin-labeled mouse monoclonal antibody to mouse CD45.1, a biotin-labeled rat monoclonal antibody to mouse CD45R/B220, a biotin-labeled rat monoclonal antibody to mouse CD19, a phycoerytrin-labeled rat monoclonal antibody to mouse CD11b, a phycoerytrin-labeled hamster monoclonal antibody to mouse CD3ε (all from BD Pharmingen), a biotin-labeled hamster monoclonal antibody to mouse TCRβ, and a phycoerytrin-labeled rat monoclonal antibody to mouse CD19 (both from eBioscience). Bone-marrow-reconstituted animals were then subjected to topic TPA treatments in the skin as indicated above.

Whereas the long-term carcinogenesis assays were done in animals of both the FVB and C57BL/10 genetic backgrounds, we selected animals of the C57BL/10 background for the short-term studies. This facilitated comparative studies with other Vav family knockout animals (i.e., single *Vav1* ^−/−^ and triple *Vav1* ^−/−^;*Vav2* ^−/−^;*Vav3* ^−/−^ mice, which were all homogenized in that background) as well as the bone marrow reconstitution experiments (which required transplantation between genetically compatible donor and recipient mice). At the beginning of each experimental procedure, cohorts of 6–8-wk-old animals with an even gender distribution of each genotype subset were used.

Samples from tumors and short-term skin experiments were processed following three independent protocols: (i) Fixed in 4% paraformaldehyde (Sigma), (ii) cryoprotected and stored at −70°C, and (iii) snap-frozen and stored at −70°C for molecular analyses (i.e., RNA and protein extraction).

### Histochemical Analyses

Tissues were extracted, fixed in 4% paraformaldehyde (Sigma), cut in 2–3 µm thick sections, and stained with hematoxylin/eosin. Tumor sections were analyzed by pathologists to classify them according to malignancy grade (benign, benign plus carcinoma in situ, malignant) and level of differentiation (high, mild poor). In short-term experiments, the thickness of the epidermal layer and total skin thickness was measured in vertical cross-sections in at least 10 different locations in each mouse to determine epidermal hyperplasia and inflammatory response (edema), respectively.

### Immunohistochemical Experiments

For standard immunohistochemical staining, paraffin-embedded sections were dewaxed, microwaved in citrate buffer, blocked with 10% nonimmune horse serum (Gibco), and incubated overnight with the appropriate primary antibody at 4°C. After exhaustive washing in 137 mM NaCl, 2.7 mM KCl, 10 mM Na_2_HPO_4_, 2 mM KH_2_PO_4_, and 0.1% Tween-20 (PBST), sections were incubated with appropriate biotin-coupled secondary antibodies (all used at a 1∶1,000 dilution in PBST) followed by avidin-peroxidase (ABC Elite Kit Vector, Vector labs). Positive staining was determined using diaminobenzidine as a substrate (DAB Kit Vector, Vector labs) following the manufacturer's recommendations. Sections were then counterstained with hematoxylin and mounted. Images were captured using an Olympus BX51 microscope coupled to an Olympus DP70 digital camera. Staining quantification was blindly assessed by two independent investigators and classified according to intensity (0–10) and estimation of ratio of cells stained (0–1). The relative value for each section was scored as the product of intensity×ratio given a general score ranging from 0 to 10. For immunofluorescent staining, sections treated as before were incubated with either a Alexa Fluor 488–labeled goat anti-rabbit IgG antibody (Molecular Probes, 1∶200 dilution) or a Cy3-conjugated goat anti-mouse IgG antibody (Jackson Immunoresearch laboratories, Inc, 1∶200 dilution), washed, and countersatined with 4′,6-diamidino-2-phenylindole (DAPI; 2 ng/ml, Sigma) with or without rhodamine-labeled phalloidin (Molecular Probes, 1∶200 dilution). Primary antibodies used in these experiments included rabbit polyclonal antibodies to keratin 1, keratin 5, keratin 14, and filaggrin (Covance, 1∶500 dilution in each case), rabbit polyclonal antibodies to IL6 (Genzyme, 1∶100 dilution), rat monoclonal antibodies to keratin 8 (Troma1, not commercial; 1/5 dilution of the hybridoma supernatant) and CD45 (BD Biosciences, 1∶50 dilution), mouse monoclonal antibodies to keratin 10 (Santa Cruz Biotechnology, 1∶50 dilution), keratin 13 (Sigma, 1∶50 dilution) and histone H2A.X (Cell Signaling, 1∶100 dilution), and a rabbit polyclonal antibody to myeloperoxidase (Abcam, 1∶250 dilution). To detect proliferating cells, dewaxed sections were denaturalized in 2N HCl at 37°C for 1 h, washed extensively in 0.1 M borate buffer, blocked with 2% nonfat dry milk (Nestlé) in 0.1% Triton-PBS, and incubated overnight with a mouse monoclonal antibody to BrdU (BD Biosciences, 1∶400 dilution). To detect apoptotic cells, we used two different approaches. In some cases, tissue sections were deparafinized, hydrated, digested with proteinase K (Dako) for 30 min at 37°C, and subjected to the Tunel reaction using the Tunel-based In Situ Cell Detection kit (Roche) as indicated by the manufacturer's instructions. Alternatively, tissues sections were cryoprotected using stepwise immersions in 15%–30% sucrose-PBS solutions, embedded in Tissue-Tek OCT (Sakura Finetek Europe), and stored frozen at −70°C. Upon thawing, slides were blocked as described before and incubated with a rabbit polyclonal antibody to cleaved caspase 3 (Cell Signaling, 1∶50 dilution). Images from immunofluoresce experiments were captured using a Leica CTR600 microscope. Quantifications of both standard and immunoflurescence signals were done with the Metamorph-Metaview software (Universal Imaging).

### Isolation and Culture of Primary Keratinocytes

Epidermis from neonates of the indicated genotypes were treated with 250 U/ml of dispase (Roche) overnight at 4°C and keratinocytes prepared using CnT07 media (CELLnTEC) according to the manufacturer's instructions. Keratinocytes were maintained in CnT07 media on type I collagen-precoated plates (BD Biosciences). When genotypically mixed cultures were utilized, the keratinocytes of one the genotypes used were labeled in culture with a cell permeable chromophore (CellTracker green CMFDA, Invitrogen) for 30 min according to the manufacturer's instructions. Labeled and nonlabeled cultures were trypsinized and plated either as genotypically pure or mixed populations in tissue culture plates.

Apoptotic rates in vitro were determined by flow cytometry using the Annexin V kit (Immunostep). In vitro cell cycle transitions were determined using the Click-iT EdU Alexa Fluor 647 Flow Cytometry Assay Kit (Invitrogen). For in vitro apoptotic assays, exponentially growing keratinocytes of the indicated genotypes were either serum starved in Eagle's minimal essential medium (EMEM, Lonza) supplemented with 0.05 mM CaCl_2_ (Sigma) or, alternatively, treated with 100 µM DMBA, 0.15 µM bleomycin (Sigma), or 2 mM dithiothreitol (Sigma). Cells were then harvested 8–24 h later and the number of apoptotic cells determined by flow cytometry using the Annexin V kit (Immunostep). In apoptotic experiments using keratinocytes ectopically expressing proteins, cells that had integrated the lentiviral particle (GFP^+^) were gated away from noninfected (GFP^−^) cells. A similar gating strategy (presence/absence of the CellTracker green CMFDA chromophore) was used to characterize apoptotic rates in genotypically mixed cell cultures. In rescue experiments with extracellular ligands, these factors were added at the beginning of the starvation period at a concentration of either 10 ng/ml (EGF, TGFα, HGF, FGF7; all obtained from Peprotech) or 100 ng/ml (IL6, Peprotech) and apoptosis quantified as above after an overnight incubation.

For keratinocyte G_1_/S phase transition assays, cells were starved for 3 h as above and then stimulated by the addition EMEM supplemented with 0.2 µM TPA, 10 ng/ml of receptors for transmembrane tyrosine kinases (EGF, TGFα HGF, FGF7), 10 ng/ml IL1β (Peprotech), 100 ng/ml IL6, or 8% calcium-chelated fetal calf serum. After 4 h, cells were incubated with the EdU reactive (Invitrogen) for 30 min, and the percentage of cells in S phase (EdU^+^) determined using the Click-iT EdU Alexa Fluor 647 Flow Cytometry Assay Kit (Invitrogen).

For intracellular signaling experiments, exponentially growing primary cells of the indicated genotypes were washed and starved for 3 h in EMEM supplemented with 0.05 mM CaCl_2_. For stimulation, we used EMEM supplemented with 0.2 µM TPA, 10 ng/ml EGF, or 100 ng/ml IL6 for the indicated periods of time. When indicated, the pan-PKC GF109203X (5 µM, Sigma), the classical PKD-specific Gö6976 (3 µM, Calbiochem), and the Src family PP2 (2 µM, Calbiochem) inhibitors were added to the starved keratinocytes 30 min before the stimulation with TPA. The PP3 molecule was used as negative control for the PP2 experiments (2 µM, Calbiochem).

When indicated, HA-Vav2, Myc-Vav3 with or without GFP were expressed in wild-type (GFP) or *Vav2* ^−/−^;*Vav3* ^−/−^ (each of the above proteins) keratinocytes using lentiviral delivery methods. To this end, the pCCM33 [17], pCCM31 [17], or pCQS1 [21] vectors were transfected into Lenti-X 293T cells (Clontech) using the Lenti-X HT packaging mix (Clontech). Viral particles were collected 48 h after transfection, concentrated using the Lenti-X concentrator kit (Clontech), and then used to infect keratinocyctes of the indicated genotypes during 3 consecutive days using centrifugation at 1,500× g in the presence of 8 µg/ml polybrene (Sigma). As controls, we carried out infections of wild-type and mutant keratinocytes using either the empty pLVX-IRES-Hyg or the GFP-encoding pcDH1-MCS1-EF1-coGFP (System Biosciences) lentivirus. The shRNA-mediated knockdown of *Fyn* transcripts was carried out using lentiviral particles (TRC lentiviral Mouse Fyn shRNA; Thermo Scientific) as previously described [17]. The TRC number and the shRNA sequence yielding the greatest knockdown was clone number TRCN0000023382 (5′-AAA CCC AGG GCT GCC TTG GAA AAG-3′). This clone was used in the experiments presented in this work.

### Protein Abundance Determinations

In the case of tissue extracts, skin samples were excised from the euthanized animals, placed on ice-cold glass plates, the epidermis removed with a razor blade, transferred into a lysis buffer (10 mM Tris-HCl [pH 8.0], 150 mM NaCl, 1% Triton X-100, 1 mM Na_3_VO_4_, 10 mM β-glycerophosphate, and a mixture of protease inhibitors [Cømplete, Roche]), and mechanically homogenized using the GentleMACS dissociator (Miltenyi Biotec). In the case of primary keratinocytes maintained in culture, cells were washed with chilled PBS, scrapped in lysis buffer, and disrupted by extensive vortexing (Ika). Extracts were precleared by centrifugation at 14,000 rpm for 10 min at 4°C, denatured by boiling in SDS-PAGE sample buffer, separated electrophoretically, and transferred onto nitrocellulose filters using the iBlot Dry Blotting System (Invitrogen). Membranes were blocked in 2% BSA (Sigma) in TBS-T (25 mM Tris-HCl (pH 8.0), 150 mM NaCl, 0.1% Tween-20) for at least 1 h and then incubated overnight with the appropriate antibodies. Those included rabbit polyclonal antibodies to phospho-Erk1/2 (residues Thr202/Tyr204; Cell Signaling, 1∶1,000 dilution), total Erk1/2 (Cell Signaling, 1∶1,000 dilution), phospho-Stat3 (residue Tyr705; Cell Signaling, 1∶1,000 dilution), total Stat3 (Cell Signaling, 1∶1,000 dilution), cyclin E (Abcam, 1∶1,000 dilution), the Myc epitope (Upstate/Millipore, 1∶1,000 dilution), Gp130 (Cell Signaling, 1∶1,000 dilution), PKC and PKD (all from Cell Signaling, 1∶1,000 dilution), as well as mouse monoclonal antibodies to α-tubulin (Calbiochem, 1∶1,000 dilution), phosphotyrosine (Santa Cruz Biotechnology, dilution 1∶1,000), the HA epitope (Covance, 1∶1,000 dilution), and IL6-Ra subunit (Santa Cruz Biotechnology, 1∶500 dilution). Homemade rabbit polyclonal antibodies to Vav2 and Vav3 have been previously described [16],[20],[24]. After three washes with TBS-T to eliminate the primary antibody, the membrane was incubated with the appropriate secondary antibody (GE Healthcare, 1∶5,000) for 30 min at room temperature. Immunoreacting bands were developed using a standard chemoluminescent method (ECL, GE Healthcare). In the case of immunoprecipitations, keratinocyte lysates obtained as above were incubated overnight at 4°C using either a rabbit polyclonal antibody to Vav2 or a monoclonal antibody to HA. Immunocomplexes were collected with Gammabind G-Sepharose beads (GE Healthcare Life Biosciences), washed, and analyzed by immunobloting as above. ELISAs were used to measure the amount of IL6 (IL6 Mouse ELISA Kit, Invitrogen) and HGF (HGF Mouse ELISA kit, Abnova) according to the manufacturer's instructions. Absorbance at 450 nm was measured immediately at the end of the protocol using a plate reader (Ultraevolution, Tecan).

### GTPase Activation Assays

Total cellular lysates were obtained as above, snap frozen, thawed, quantified, and analyzed using Rac1 and RhoA G-LISA assay kits according to the manufacturer's instructions (Cytoskeleton). Absorbance at 490 nm was measured using a Ultraevolution plate reader.

### Microarray Analyses

All microarray experiments were performed by the personnel of the Genomics and Proteomics Unit of our Institution. Total cellular RNA was extracted from the back skin of wild-type or knockout mice treated as described before (*n* = 3 for each treatment) using the RNAeasy kit (Qiagen), quantified using 6000 Nano Chips (Agilent), and used (2.3 µg/sample) to generate labeled cRNA probes according to the manufacturer's instructions (Affymetrix). Purified RNA was processed and hybridized to Affymetrix GeneChip Mouse Gene 1.0 ST as indicated elsewhere [17],[50]. Signal intensity values were obtained from CEL files after robust multichip average [51],[52]. We performed Pavlidis template matching analysis, in which the Pearson's correlation coefficient is computed between the intensities measured for each gene and the values of an independent variable [53]. The *p* values to test for the null hypothesis that the correlation is zero are provided. Corrected *p* values were also calculated (*Q* value). For functional annotation purposes, genes were considered differentially regulated if their *Q* values were lower than 0.05. For metagenomic analysis, we used as threshold significance values a *Q*≤0.025 and fold change variations relative to control skin ≥1.5. Graphical representation of microarray data was generated using hierarchical clustering analysis and the BioConductor HCLUST tool.

Further bioinformatics were used to check the expression of Vav2/Vav3-dependent mRNAs in normal skin and skin tumors. To that end, probesets within GSE21264 dataset corresponding to A_1_ and A_2_ gene clusters were analyzed from differential expression between normal tail, papillomas, and carcinomas using the microarray database indicated above and ANOVA analysis (*p*≤0.01). For those genes with more than one probeset significantly deregulated, we selected the one with the lowest *p* value for representation.

The raw microarray data generated in this work have been uploaded to the GEO database (www.ncbi.nlm.nih.gov/geo/query/acc.cgi?token=pjcxliqugwowqfq&acc=GSE40849).

### Statistics

Differences in tumor multiplicity and incidence were analyzed by the Mann-Whitney U test and the χ^2^ test, respectively. Other wet lab data were processed using the Student's *t* or the one tail Mann-Whitney tests. In all cases, *p* values lower than 0.05 were considered statistically significant. *p* values were represented in all figures as * (when ≤0.05), ** (when ≤0.01), and *** (when ≤0.001). Data obtained are given as the mean ± the s.e.m.

## Supporting Information

Figure S1 Expression of Vav family members and Rho GEFs of the Dbl family in CSTs. (A, B) qRT-PCR determination of the number of copies (A) and relative abundance (B) of *Vav* family mRNAs in keratinocytes (A), normal skin (B), and DMBA/TPA-induced papillomas (B) from FVB (A, B) and C57BL/10 mice (A). In (B), values are given relative to the abundance of the indicated transcript in the appropriate control sample (which was given an arbitrary value of 1). au, arbitrary units. These results show that the *Vav2* and *Vav3* mRNAs are expressed at much higher levels than the *Vav1* transcript in primary keratinocytes isolated from mice of both the FVB or C57BL/10 genetic backgrounds. The abundance of those two transcripts increased about 3-fold in DMBA/TPA-induced papillomas and cutaneous squamous cell caracinomas (cSCCs) when compared to normal skin. By contrast, the *Vav1* mRNA only underwent a significant increase in abundance in cSCCs, although its overall expression levels remained significantly lower than those for the other two Vav family transcripts using copy number criteria. (C) Bioinformatic analysis of the expression pattern of Rho/Rac GEF-encoding mRNAs in skin and CSTs in the Balmain's dataset. This dataset was generated with Affymetrix microarrays using samples from DMBA/TPA-induced tumors and paired normal tail skin samples [38]. Signal log ratio abundance levels are depicted as gradients from dark blue (lowest abundance) to dark red (highest abundance). Sample type is indicated at the top. Clusters (C1–C4) of similarly expressed mRNAs are indicated on the left. Transcript names are indicated on the right, with *Vav* family and *Tiam1* mRNAs highlighted in red. These data indicate that, in addition to the *Vav* family mRNAs and the previously described *Tiam1* transcript [4], 51 additional Rho/Rac GEFs are also enriched in skin (cluster C1), skin and papilloma (cluster C2), papillomas and cSCCs (cluster C3), or cSCCs (cluster C4).
(TIF)

Figure S2 Analysis of the skin of wild-type and *Vav2* ^−/−^;*Vav3* ^−/−^ mice. (A) Hematoxylin-eosin stained skin sections from newborn mice of the indicated genotypes (top) and postnatal stages (P, left). Scale bar, 100 µm (*n* = 3 mice of each genotype). *WT*, wild type; *DKO*, double *Vav2* ^−/−^;*Vav3* ^−/−^ knockout mice. (B) Expression of the indicated differentiation keratinocyte markers (left) in the dorsal back skin of adult mice of the indicated genotypes (top) (*n* = 3 mice of each genotype). In addition to antibodies to keratin K14 (K14), keratin K1 (K1, ID number: 16678), and filaggrin (signals shown in green color), sections were counterstained with 4′,6-diamidino-2-phenylindole (DAPI) and rhodamine-phalloidin to visualize cell nuclei (blue color) and F-actin (red color), respectively. Scale bar, 100 µm. These results show that the histological structure and differentiation process are quite similar in skin of all animals analyzed.
(TIF)

Figure S3 Normal levels of Erk and Rac1 activation are restored in *Vav2* ^−/−^;*Vav3* ^−/−^ keratinocytes upon the ectopic expression of Vav family proteins. (A, B) Phosphorylation and expression status of Erk1,2 in serum-starved wild-type and *Vav2* ^−/−^;*Vav3* ^−/−^ keratinocytes that were transduced with empty (A, B), HA-Vav2 (A), and Myc-Vav3-encoding (B) lentiviruses prior to starvation and subsequent TPA stimulation for the indicated periods of time (*n* = 3). (C) Rac1 activity levels induced by TPA in quiescent wild-type and *Vav2* ^−/−^;*Vav3* ^−/−^ keratinocytes that were transduced prior to the stimulation step with the indicated lentiviruses (*n* = 3).
(TIF)

Figure S4 Dissection of the TPA/Vav-dependent route in keratinocytes. (A) Expression and TPA-induced activation of indicated PKC family proteins and the protein kinase D (PKD) substrate in total cellular lysates obtained from wild-type keratinocytes upon a 5 min stimulation with either TPA or EGF (top) (*n* = 1). MW, molecular weight. (B) qRT-PCR determination of the abundance of the *Fyn* mRNA in wild-type keratinocytes that were either mock infected or transduced with lentiviruses encoding a Fyn-specific shRNA (*n* = 3). Values are given relative to the abundance of the transcript in mock-infected cells (which was given an arbitrary value of 1).
(TIF)

Figure S5 Vav proteins regulate the expression of a large transcriptomal program during the promotion phase of skin tumors. (A) Hierarchical cluster diagram of mRNAs whose abundance changes more than 1.5-fold in a Vav2/Vav3-independent (left panel) or -dependent (right panel) manner between control and TPA-stimulated skin. Signal log ratio abundance levels are depicted on a dark blue (lowest abundance) to dark red (highest abundance) scale. Columns represent replicates of the indicated experimental groups. (B) In silico analysis of the expression of Vav2/Vav3-dependent genes belonging to the A_1_ (top panel) and A_2_ (bottom panel) clusters in normal mouse skin and in DMBA/TPA-induced mouse papillomas and skin carcinomas (red). mRNA abundance is depicted as in (A). Specific cluster expression groups are indicated at the right. (C, D) qRT-PCR analysis showing the abundance of indicated mRNAs in naïve skin (C), TPA-stimulated skin (C), quiescent keratinocytes (D), and TPA-stimulated keratinocytes (D) of indicated genotypes (*n* = 3).
(TIF)

Figure S6 Vav proteins promote autocrine/paracrine signaling in TPA-stimulated skin. (A) ELISA detection of HGF (top panels) and IL6 (bottom panels) in the skin (left panels) and serum (right panels) 24 h upon the stimulation of mice of indicated genotypes with TPA (*n* = 3). (B) Immunohistochemical detection of IL6 (brown color) in the epidermis of mice of indicated genotypes after being treated with either acetone or TPA for 24 h (*n* = 5).
(TIF)

Figure S7 Implication of Fyn in the keratinocyte TPA/Vav-dependent autocrine/paracrine program. qRT-PCR-determined abundance of indicated mRNAs in control and *Fyn*-knockdown keratinocytes (both from wild-type mice) upon stimulation with TPA for indicated periods of time (*n* = 3).
(TIF)

Figure S8 The expression of Vav-dependent genes of breast cancer cells is also suppressed in the epidermis of TPA-stimulated *Vav2* ^−/−^;*Vav3* ^−/−^ mice. (A, B) qRT-PCR analysis of the expression of indicated genes in TPA-stimulated epidermis (A) and keratinocytes (B) obtained from mice of indicated genotypes (*n* = 3). Values are given relative to the abundance of each indicated transcript in the control sample (which was given an arbitrary value of 1).
(TIF)

Figure S9 The *Vav2/Vav3* gene deficiency does not affect the expression of the IL6 receptor. (A, B) qRT-PCR (A, *n* = 3) and immunoblot (B; *n* = 2) analyses showing the abundance of indicated IL6 receptor subunits (α [ID number: 16194], glycoprotein 130 [Gp130, ID number: 16195]) in the epidermis (A) and cultured keratinocytes (A, B) obtained from mice of indicated genotypes. *Il6st* is the transcript for the Gp130 IL6-R subunit.
(TIF)

Table S1 Histological analysis of skin tumors developed in FVB mice of the indicated genotypes using the DMBA+TPA treatment.
(DOCX)

Table S2 Histological analysis of skin tumors developed in FVB mice of the indicated genotypes using the DMBA/DMBA treatment.
(DOCX)

Table S3 Microarray analysis of the Vav2/Vav3-dependent transcriptome in skin.
(XLS)

Table S4 Examples of Vav2/Vav3-dependent extracellular factors induced during the promotion phase. Genes up-regulated or down-regulated in the skin of TPA-stimulated wild-type but not in *Vav2* ^−/−^;*Vav3* ^−/−^ mice are shown in red and blue, respectively. Selected genes have been included according to statistical significance value and independently of any fold change variation criteria.
(DOC)

## Abbreviations

Areg: amphiregulin
Asef: adenomatous polyposis coli-stimulated exchange factor 1
BM: bone marrow
BrdU: Bromodeoxyuridine
cPKC: classical protein kinase C
cSCC: cutaneous squamous cell carcinoma
CST: cutaneous squamous tumor
DAPI: 4′,6-diamidino-2-phenylindole
DH: Dbl-homology
DMBA: 7,12-dimethylben[*a*]antracene
Dock: dedicator of cytokinesis
EdU: 5-ethynyl-2′deoxyuridine
EGF: epidermal growth factor
ELISA: enzyme-linked immunosorbent assay
Erk: extracellular-regulated kinase
ET: epidermal thickness
FGF: fibroblast growth factor
GEF: guanosine nucleotide exchange factor
GFP: green fluorescent protein
G-LISA: GTPase-linked immunosorbent assay
Gp130: Glycoprotein 130
GTPase: GTP hydrolase
HA: hemagglutinin epitope
HbEGF: heparin-binding EGF-like growth factor
HGF: hepatocyte growth factor
IL: interleukin
IL6-R: IL6 receptor
NFAT: nuclear factor of activated cells
NFκB: nuclear factor kappa-light-chain-enhancer of activated B cells
PKC: protein kinase C
qRT-PCR: quantitative reverse transcription-polymerase chain reaction
P-Rex1: phosphatidylinositol 3,4,5-triphosphate-dependent Rac exchanger 1
shRNA: short hairpin RNA
Stat: Signal transducer and activator of transcription
TPA: 12-O-tetradecanoylphorbol-13-acetate
TGF: tumor growth factor
T_H_: T helper lymphocyte
Tiam1: T-cell lymphoma invasion and metastasis-inducing protein 1
TKO: triple *Vav1* ^−/−^
*Vav2*^−/−^
*Vav3*^−/−^: knockout mice
TST: total skin thickness
Tunel: terminal deoxynucleotidyl transferase dUTP nick end labelling; VEGF, vascular endothelial growth factor
WT: wild type
