# Supplementary figures and images for: The Rho Exchange Factors Vav2 and Vav3 Favor Skin Tumor Initiation and Promotion by Engaging Extracellular Signaling Loops
Source: PLoS Biol. 2013 Jul 23;11(7):e1001615. doi: 10.1371/journal.pbio.1001615 (PMC3720258; doi:10.1371/journal.pbio.1001615)

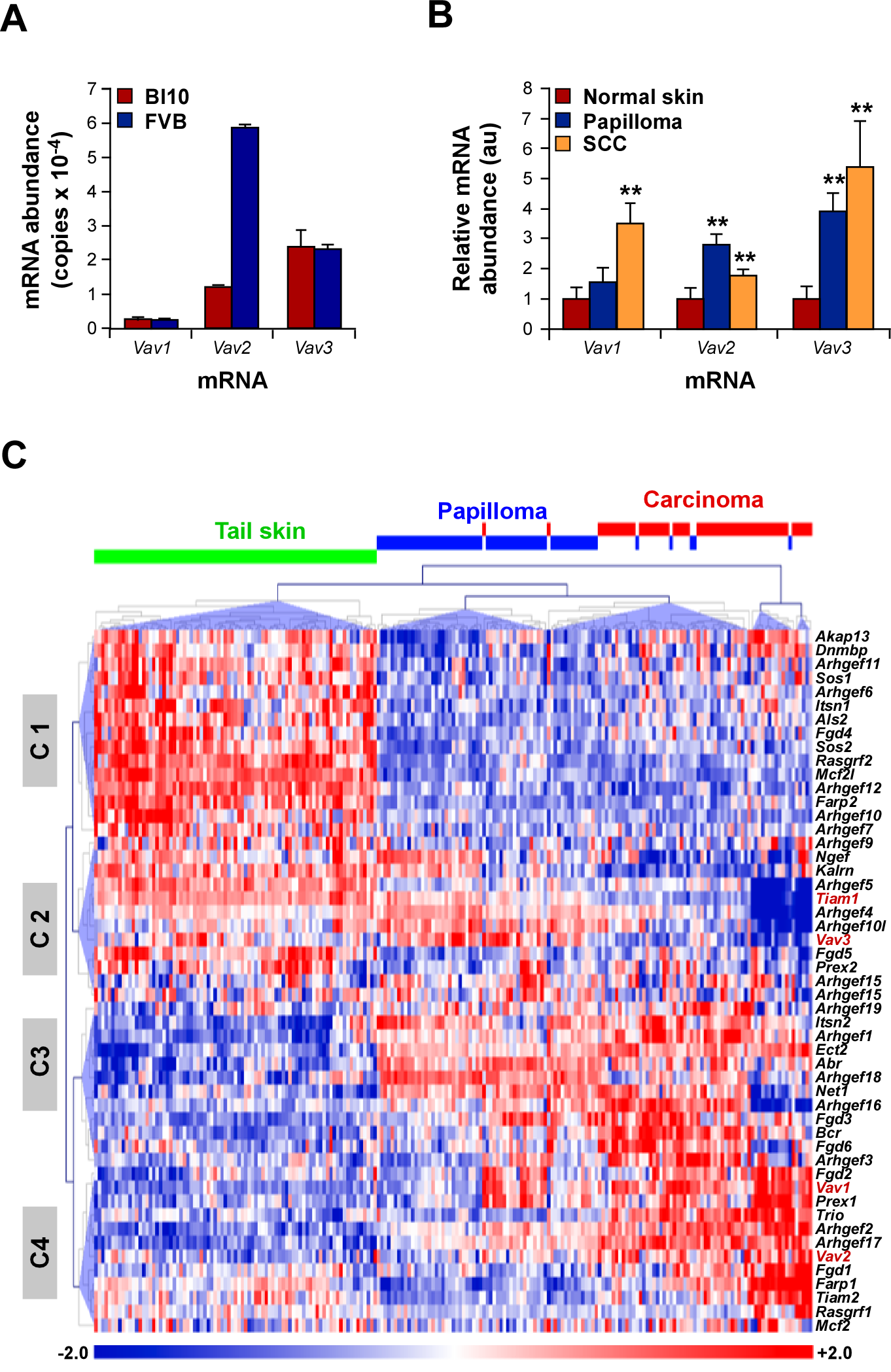

Supplement: Figure S1 — Expression of Vav family members and Rho GEFs of the Dbl family in CSTs. (A, B) qRT-PCR determination of the number of copies (A) and relative abundance (B) of Vav family mRNAs in keratinocytes (A), normal skin (B), and DMBA/TPA-induced papillomas (B) from FVB (A, B) and C57BL/10 mice (A). In (B), values are given relative to the abundance of the indicated transcript in the appropriate control sample (which was given an arbitrary value of 1). au, arbitrary units. These results show that the Vav2 and Vav3 mRNAs are expressed at much higher levels than the Vav1 transcript in primary keratinocytes isolated from mice of both the FVB or C57BL/10 genetic backgrounds. The abundance of those two transcripts increased about 3-fold in DMBA/TPA-induced papillomas and cutaneous squamous cell caracinomas (cSCCs) when compared to normal skin. By contrast, the Vav1 mRNA only underwent a significant increase in abundance in cSCCs, although its overall expression levels remained significantly lower than those for the other two Vav family transcripts using copy number criteria. (C) Bioinformatic analysis of the expression pattern of Rho/Rac GEF-encoding mRNAs in skin and CSTs in the Balmain's dataset. This dataset was generated with Affymetrix microarrays using samples from DMBA/TPA-induced tumors and paired normal tail skin samples [38]. Signal log ratio abundance levels are depicted as gradients from dark blue (lowest abundance) to dark red (highest abundance). Sample type is indicated at the top. Clusters (C1–C4) of similarly expressed mRNAs are indicated on the left. Transcript names are indicated on the right, with Vav family and Tiam1 mRNAs highlighted in red. These data indicate that, in addition to the Vav family mRNAs and the previously described Tiam1 transcript [4], 51 additional Rho/Rac GEFs are also enriched in skin (cluster C1), skin and papilloma (cluster C2), papillomas and cSCCs (cluster C3), or cSCCs (cluster C4). (TIF) [file pbio.1001615.s001.tif]

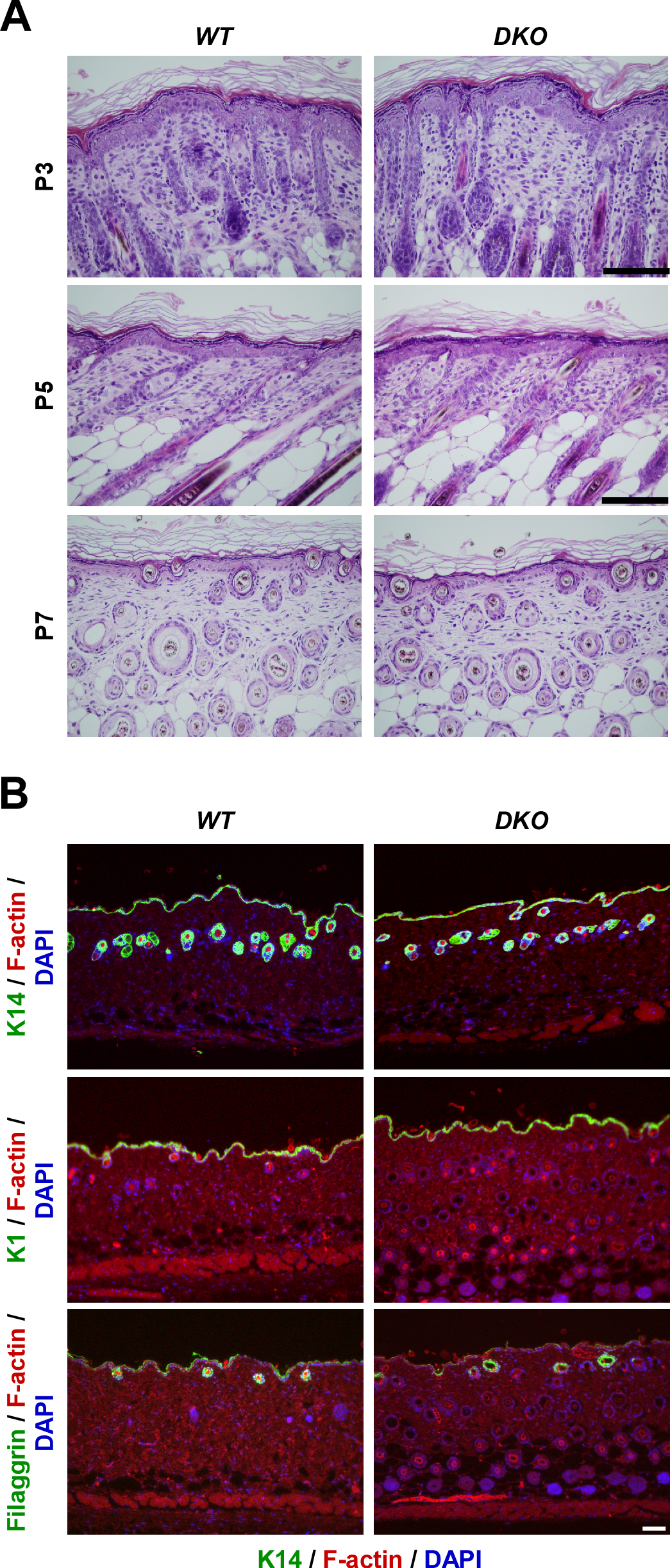

Supplement: Figure S2 — Analysis of the skin of wild-type and Vav2 −/−;Vav3 −/− mice. (A) Hematoxylin-eosin stained skin sections from newborn mice of the indicated genotypes (top) and postnatal stages (P, left). Scale bar, 100 µm (n = 3 mice of each genotype). WT, wild type; DKO, double Vav2 −/−;Vav3 −/− knockout mice. (B) Expression of the indicated differentiation keratinocyte markers (left) in the dorsal back skin of adult mice of the indicated genotypes (top) (n = 3 mice of each genotype). In addition to antibodies to keratin K14 (K14), keratin K1 (K1, ID number: 16678), and filaggrin (signals shown in green color), sections were counterstained with 4′,6-diamidino-2-phenylindole (DAPI) and rhodamine-phalloidin to visualize cell nuclei (blue color) and F-actin (red color), respectively. Scale bar, 100 µm. These results show that the histological structure and differentiation process are quite similar in skin of all animals analyzed. (TIF) [file pbio.1001615.s002.tif]

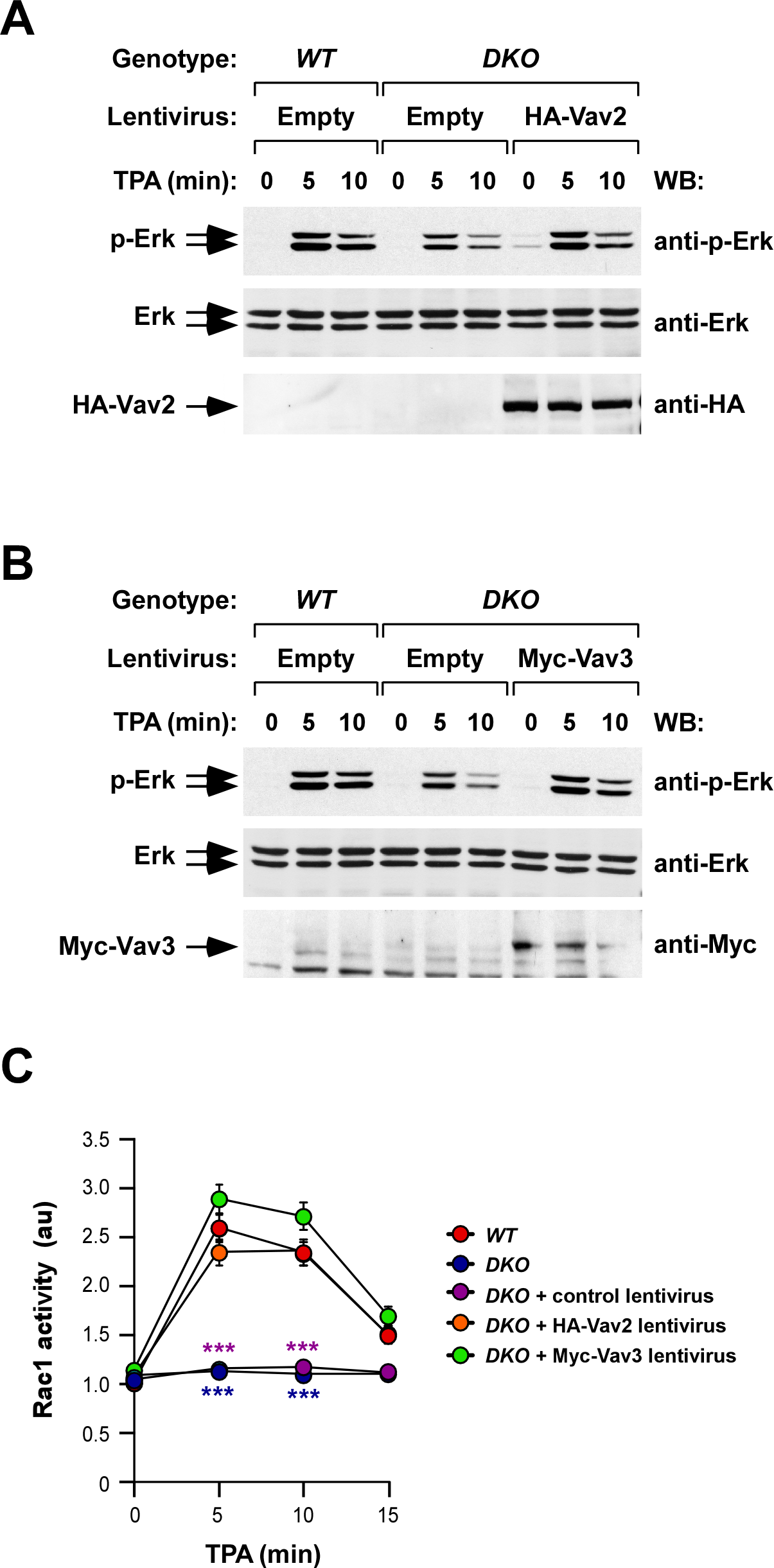

Supplement: Figure S3 — Normal levels of Erk and Rac1 activation are restored in Vav2 −/−;Vav3 −/− keratinocytes upon the ectopic expression of Vav family proteins. (A, B) Phosphorylation and expression status of Erk1,2 in serum-starved wild-type and Vav2 −/−;Vav3 −/− keratinocytes that were transduced with empty (A, B), HA-Vav2 (A), and Myc-Vav3-encoding (B) lentiviruses prior to starvation and subsequent TPA stimulation for the indicated periods of time (n = 3). (C) Rac1 activity levels induced by TPA in quiescent wild-type and Vav2 −/−;Vav3 −/− keratinocytes that were transduced prior to the stimulation step with the indicated lentiviruses (n = 3). (TIF) [file pbio.1001615.s003.tif]

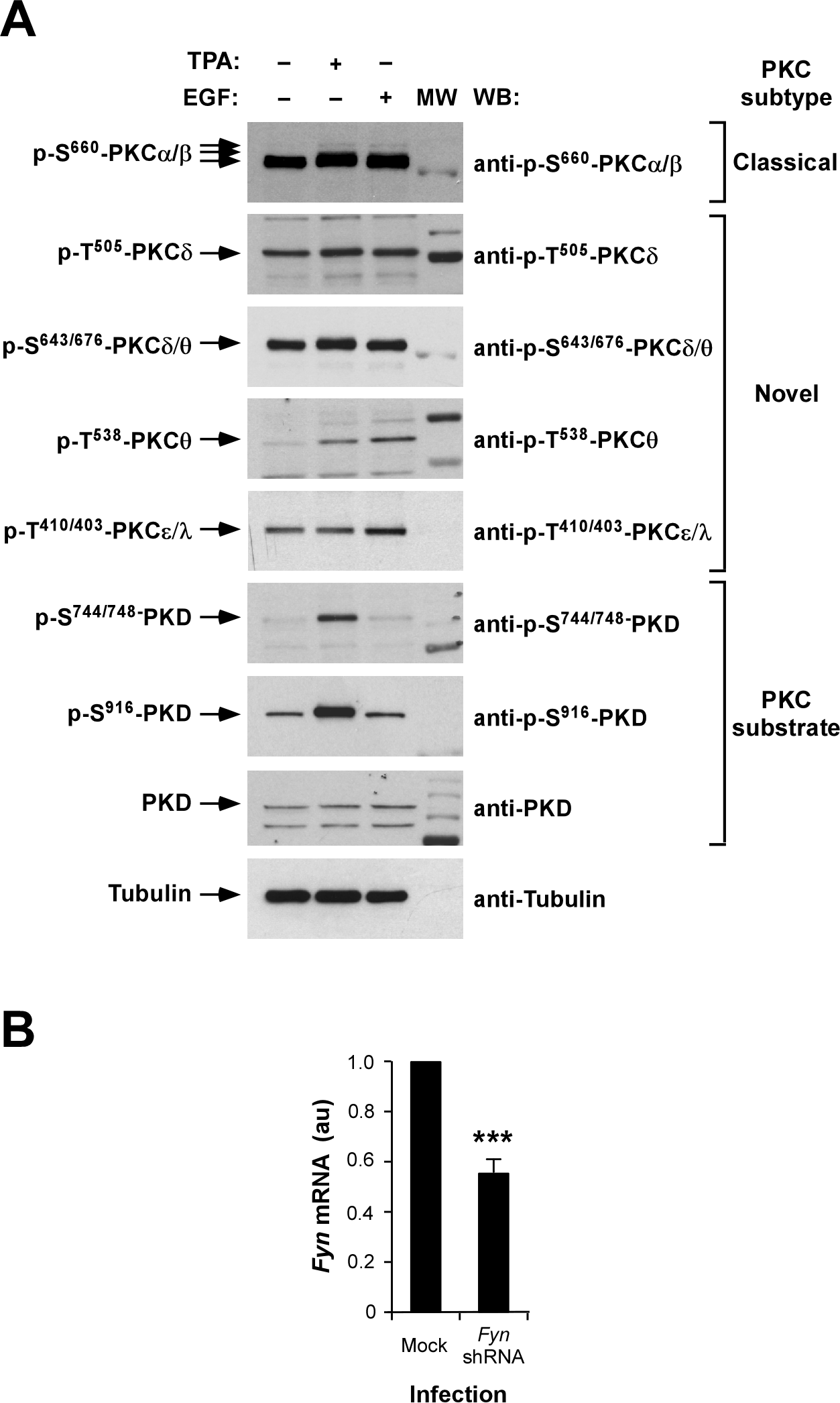

Supplement: Figure S4 — Dissection of the TPA/Vav-dependent route in keratinocytes. (A) Expression and TPA-induced activation of indicated PKC family proteins and the protein kinase D (PKD) substrate in total cellular lysates obtained from wild-type keratinocytes upon a 5 min stimulation with either TPA or EGF (top) (n = 1). MW, molecular weight. (B) qRT-PCR determination of the abundance of the Fyn mRNA in wild-type keratinocytes that were either mock infected or transduced with lentiviruses encoding a Fyn-specific shRNA (n = 3). Values are given relative to the abundance of the transcript in mock-infected cells (which was given an arbitrary value of 1). (TIF) [file pbio.1001615.s004.tif]

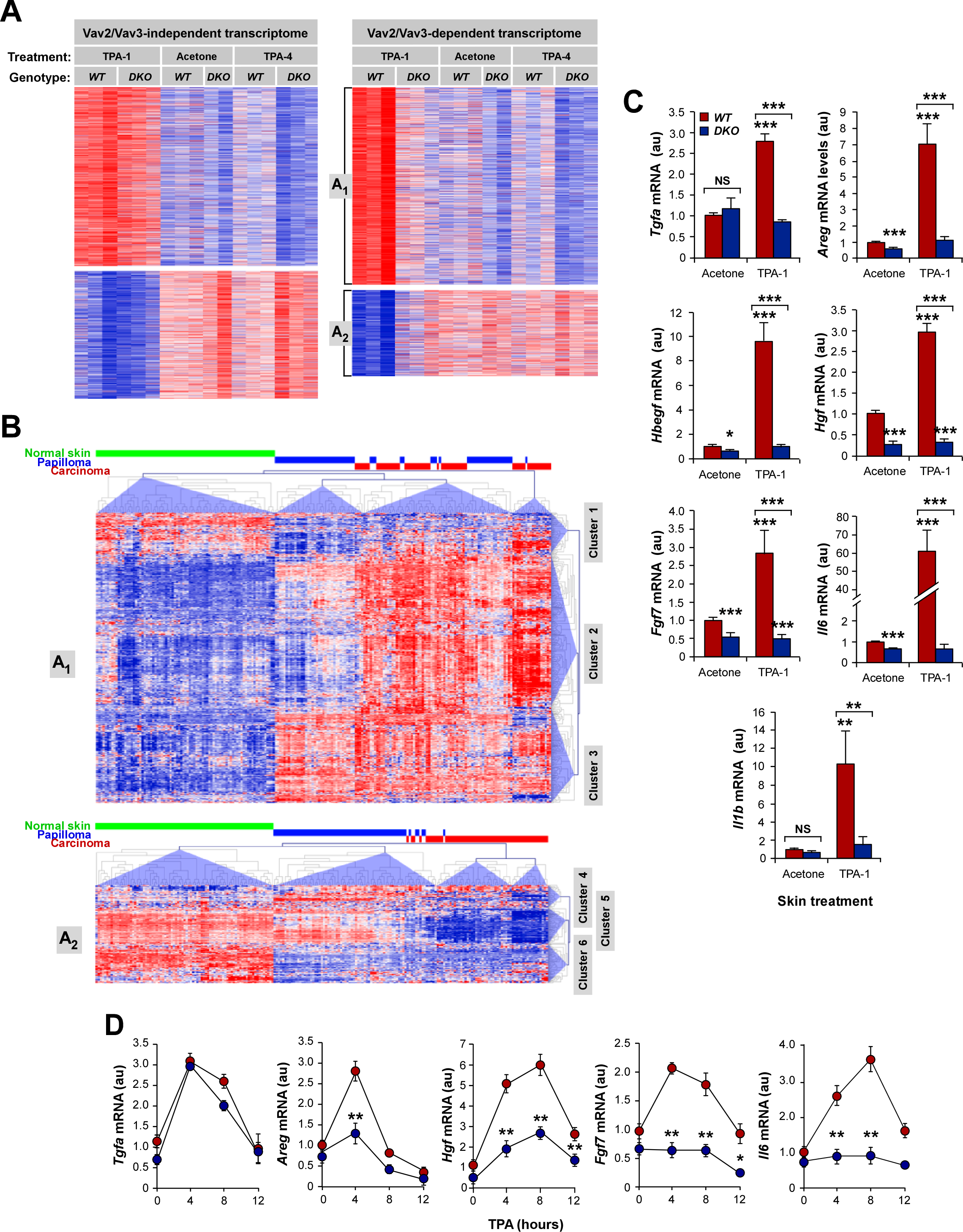

Supplement: Figure S5 — Vav proteins regulate the expression of a large transcriptomal program during the promotion phase of skin tumors. (A) Hierarchical cluster diagram of mRNAs whose abundance changes more than 1.5-fold in a Vav2/Vav3-independent (left panel) or -dependent (right panel) manner between control and TPA-stimulated skin. Signal log ratio abundance levels are depicted on a dark blue (lowest abundance) to dark red (highest abundance) scale. Columns represent replicates of the indicated experimental groups. (B) In silico analysis of the expression of Vav2/Vav3-dependent genes belonging to the A1 (top panel) and A2 (bottom panel) clusters in normal mouse skin and in DMBA/TPA-induced mouse papillomas and skin carcinomas (red). mRNA abundance is depicted as in (A). Specific cluster expression groups are indicated at the right. (C, D) qRT-PCR analysis showing the abundance of indicated mRNAs in naïve skin (C), TPA-stimulated skin (C), quiescent keratinocytes (D), and TPA-stimulated keratinocytes (D) of indicated genotypes (n = 3). (TIF) [file pbio.1001615.s005.tif]

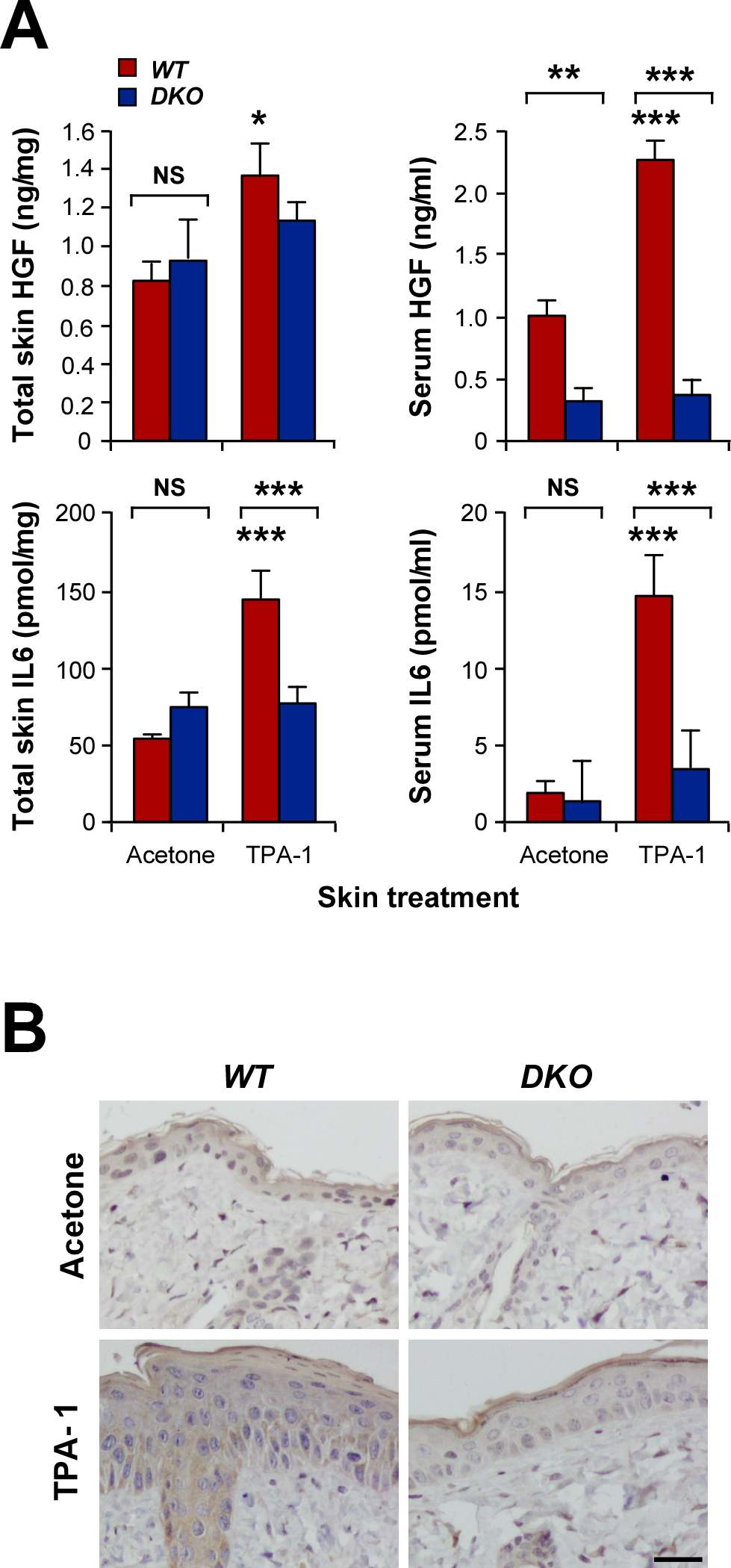

Supplement: Figure S6 — Vav proteins promote autocrine/paracrine signaling in TPA-stimulated skin. (A) ELISA detection of HGF (top panels) and IL6 (bottom panels) in the skin (left panels) and serum (right panels) 24 h upon the stimulation of mice of indicated genotypes with TPA (n = 3). (B) Immunohistochemical detection of IL6 (brown color) in the epidermis of mice of indicated genotypes after being treated with either acetone or TPA for 24 h (n = 5). (TIF) [file pbio.1001615.s006.tif]

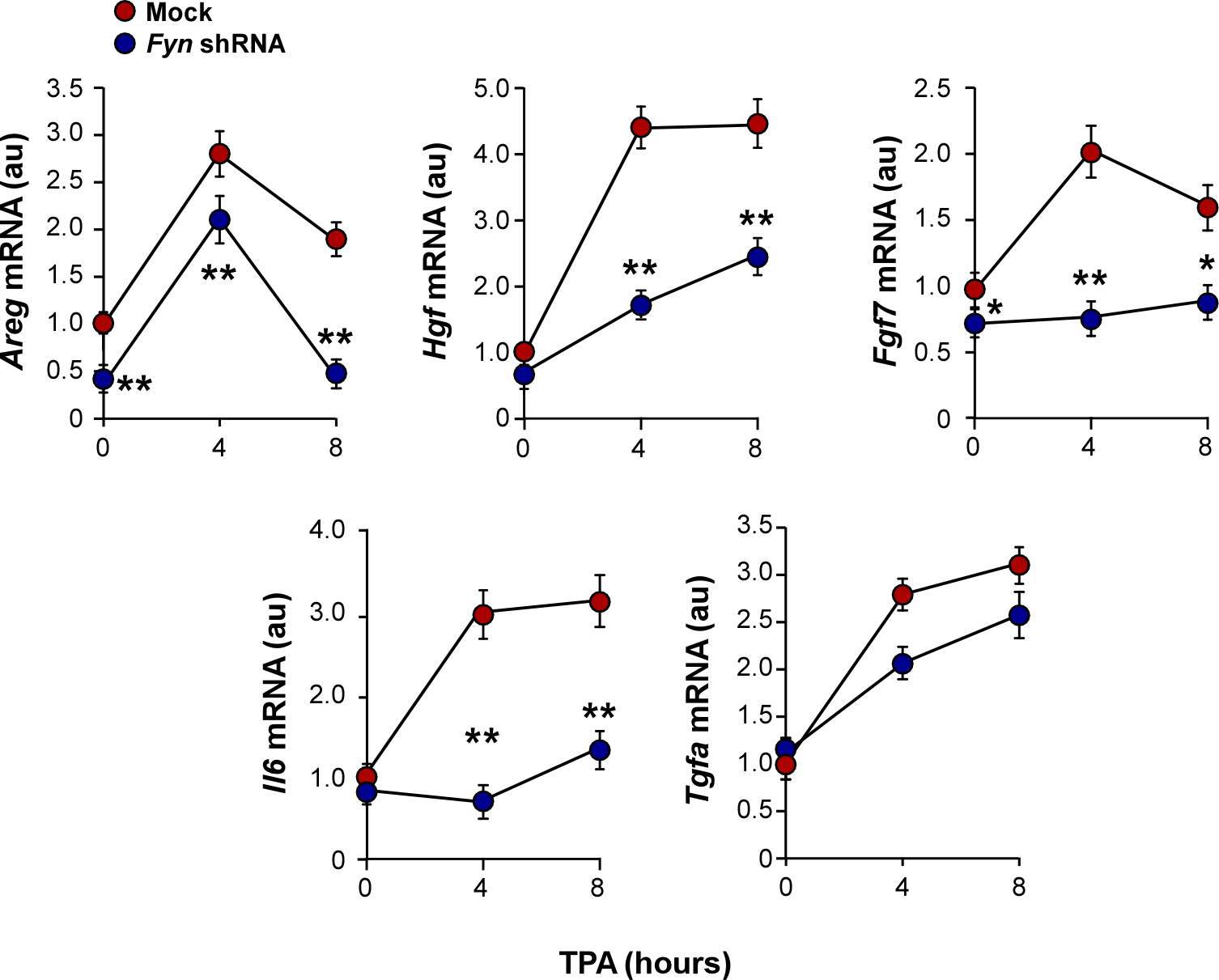

Supplement: Figure S7 — Implication of Fyn in the keratinocyte TPA/Vav-dependent autocrine/paracrine program. qRT-PCR-determined abundance of indicated mRNAs in control and Fyn-knockdown keratinocytes (both from wild-type mice) upon stimulation with TPA for indicated periods of time (n = 3). (TIF) [file pbio.1001615.s007.tif]

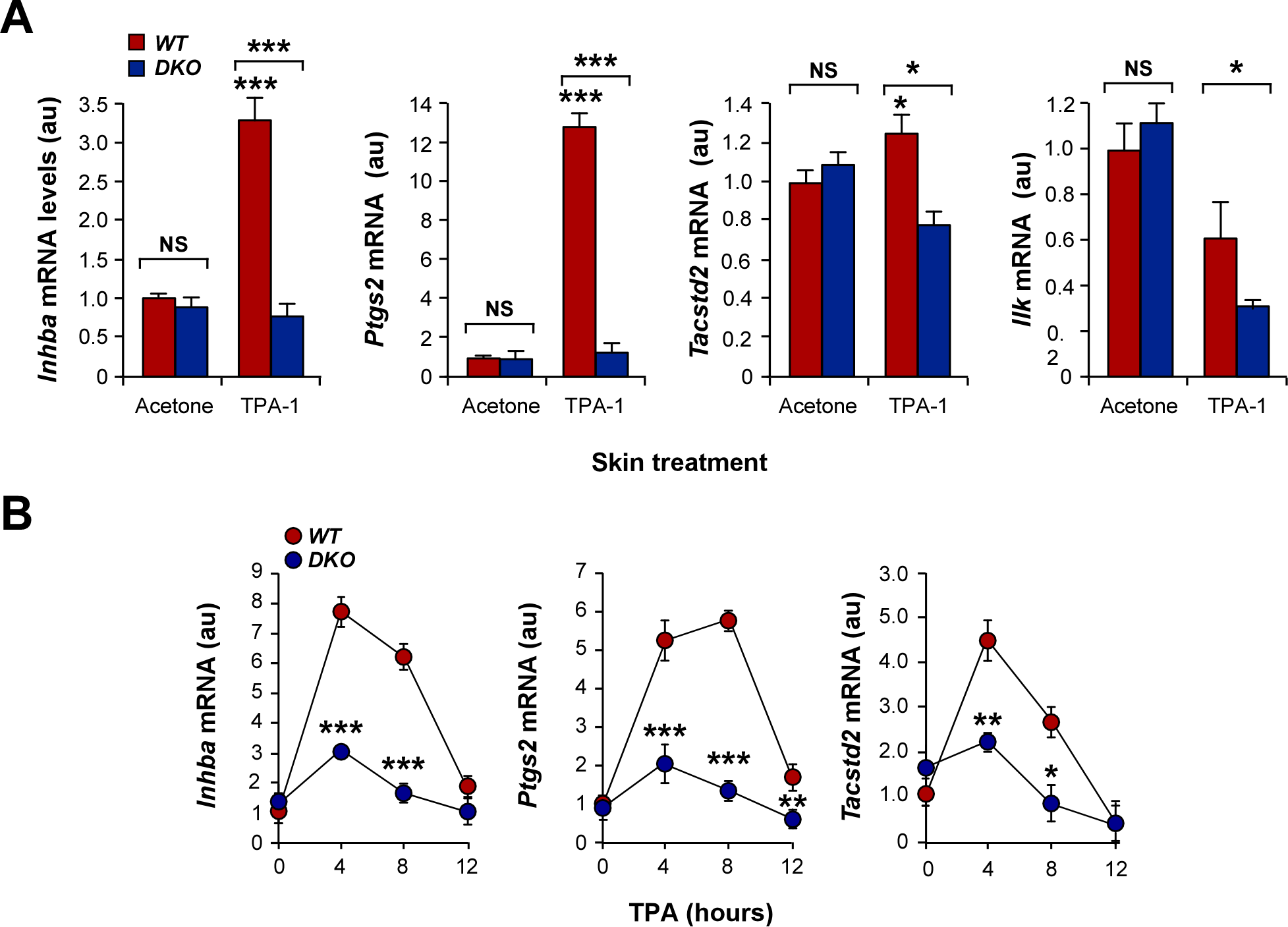

Supplement: Figure S8 — The expression of Vav-dependent genes of breast cancer cells is also suppressed in the epidermis of TPA-stimulated Vav2 −/−;Vav3 −/− mice. (A, B) qRT-PCR analysis of the expression of indicated genes in TPA-stimulated epidermis (A) and keratinocytes (B) obtained from mice of indicated genotypes (n = 3). Values are given relative to the abundance of each indicated transcript in the control sample (which was given an arbitrary value of 1). (TIF) [file pbio.1001615.s008.tif]

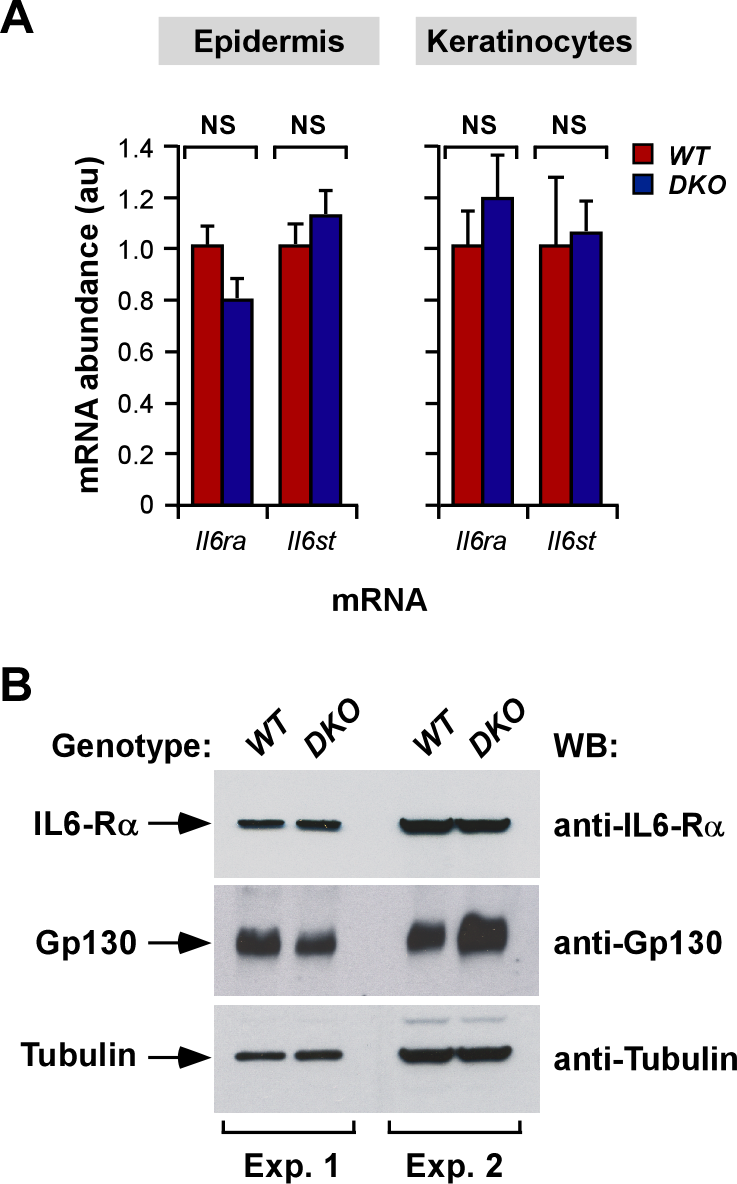

Supplement: Figure S9 — The Vav2/Vav3 gene deficiency does not affect the expression of the IL6 receptor. (A, B) qRT-PCR (A, n = 3) and immunoblot (B; n = 2) analyses showing the abundance of indicated IL6 receptor subunits (α [ID number: 16194], glycoprotein 130 [Gp130, ID number: 16195]) in the epidermis (A) and cultured keratinocytes (A, B) obtained from mice of indicated genotypes. Il6st is the transcript for the Gp130 IL6-R subunit. (TIF) [file pbio.1001615.s009.tif]
