# Supplementary material for: The Rho Exchange Factors Vav2 and Vav3 Favor Skin Tumor Initiation and Promotion by Engaging Extracellular Signaling Loops
Source: PLoS Biol. 2013 Jul 23;11(7):e1001615. doi: 10.1371/journal.pbio.1001615 (PMC3720258; doi:10.1371/journal.pbio.1001615)
Supplement: Table S1 — Histological analysis of skin tumors developed in FVB mice of the indicated genotypes using the DMBA+TPA treatment. (DOCX) [file pbio.1001615.s010.docx]

**TABLE S1.** Histological analysis of skin tumors developed in FVB mice of the indicated genotypes using the DMBA+TPA treatment.

|  | ***WT*** | | ***Vav2*^–/–^;*Vav3*^–/–^** | |
| --- | --- | --- | --- | --- |
| **TUMOR TYPE** | ***n^a^*** | **%** | ***n*** | **%** |
| **Benign lesions** | **25** | **65.79** | **29** | **65.91** |
| Papilloma | 16 | 42.11 | 28 | 63.64 |
| Carcinoma *in situ* | 9 | 23.37 | 1 | 2.27^b^ |
|  |  |  |  |  |
| **Malignant lesions** | **13** | **34.21** | **15** | **34.09** |
| High differentiation | 9 | 23.68 | 11 | 25 |
| Mild differentiation | 3 | 7.89 | 2 | 4.55 |
| Poor differentiation | 1 | 2.63 | 2 | 4.55 |

^a^Numbers refer to the total number of tumors analyzed, not the number of tumors developing per mouse.

^b^Statistically significant variation relative to the *WT* control (*P* ≤ 0.01) using a χ^2^ test.
