# Supplementary material for: The Rho Exchange Factors Vav2 and Vav3 Favor Skin Tumor Initiation and Promotion by Engaging Extracellular Signaling Loops
Source: PLoS Biol. 2013 Jul 23;11(7):e1001615. doi: 10.1371/journal.pbio.1001615 (PMC3720258; doi:10.1371/journal.pbio.1001615)
Supplement: Table S2 — Histological analysis of skin tumors developed in FVB mice of the indicated genotypes using the DMBA/DMBA treatment. (DOCX) [file pbio.1001615.s011.docx]

**TABLE S2.** Histological analysis of skin tumors developed in FVB mice of the indicated genotypes using the DMBA/DMBA treatment.

|  | ***WT*** | | ***Vav2*^–/–^;*Vav3*^–/–^** | |
| --- | --- | --- | --- | --- |
| **TUMOR TYPE** | ***n^a^*** | **%** | ***n*** | **%** |
| **Benign lesions** | **2** | **9.52** | **7** | **25.00^b^** |
| Papilloma | 1 | 4.76 | 4 | 14.29 |
| Carcinoma in situ | 1 | 4.76 | 3 | 10.71 |
|  |  |  |  |  |
| **Malignant lesions** | **19** | **90.48** | **21** | **75.00^b^** |
| High differentiation | 10 | 47.62 | 15 | 53.57 |
| Mild differentiation | 6 | 28.57 | 3 | 10.71 |
| Poor differentiation | 3 | 14.29 | 3 | 10.71 |

^a^Numbers refer to the total number of tumors analyzed, not the number of tumors developing per mouse.

^b^Statistically significant variation relative to the *WT* control (*P* ≤ 0.05) using a χ^2^ test.
