# Supplementary material for: The Rho Exchange Factors Vav2 and Vav3 Favor Skin Tumor Initiation and Promotion by Engaging Extracellular Signaling Loops
Source: PLoS Biol. 2013 Jul 23;11(7):e1001615. doi: 10.1371/journal.pbio.1001615 (PMC3720258; doi:10.1371/journal.pbio.1001615)
Supplement: Table S4 — Examples of Vav2/Vav3-dependent extracellular factors induced during the promotion phase. Genes up-regulated or down-regulated in the skin of TPA-stimulated wild-type but not in Vav2 −/−;Vav3 −/− mice are shown in red and blue, respectively. Selected genes have been included according to statistical significance value and independently of any fold change variation criteria. (DOC) [file pbio.1001615.s013.doc]

**TABLE S4.** Examples of Vav2/Vav3-dependent extracellular factors induced during the promotion phase. Genes upregulated or downregulated in the skin of TPA-stimulated wild type but not in *Vav2*^–/–^;*Vav3*^–/–^ mice are shown in red and blue, respectively. Genes also upregulated in a Vav2/Vav3-dependent manner in breast cancer cells are underscored (see main text). Selected genes have been included according to statistical significance value and independently of any fold change variation criteria.

| **For transmembrane kinase receptors** | **Cytokines** |
| --- | --- |
| *Angpt2* | *Il6* |
| *Areg* | *Il10* |
| *Ereg* | *Il15* |
| *Fgf23* | *Il16* |
| *Fgf7* | *Il17b* |
| *Flt3l* | *Il23a* |
| *Hbegf* | *Il18* |
| *Hgf* | *Il19* |
| *Ngf* | *Il21* |
| *Nrg1* | *Il24* |
| *Nrg4* | *Il33* |
| *Pdgfa* | *Lif* |
| *Pdgfb* | *Ltb* |
| *Tgfa* | *Osm* |
| *Tgfb1* | *Tnf* |
| *Tgfb2* |  |
| *Tgfbi* | **T_H1_-related proteins** |
| *Vegfb* | *Ccl3* |
| **Chemokines** | *Ccl4* |
| *Ccl2* | *Ccr1* |
| *Ccl3* | *Ccr5* |
| *Ccl4* | *Cxcl5* |
| *Ccl6* | *Il1b* |
| *Ccl7* | *Spp1* |
| *Ccl8* | *Tnfa* |
| *Ccl9* |  |
| *Ccl11* | **T_H2_-related proteins** |
| *Cxcl1* | *Ccl11* |
| *Cxcl2* |  |
| *Cxcl3* | **T_H17_-related proteins** |
| *Cxcl5* | *Clec7a* |
| *Cxcl12* | *Csf3* |
| *Cxcl13* | *Cxcl1* |
| *Cxcl14* | *Cxcl2* |
| **Cytokines** | *Cxcl5* |
| *Csf3* | *Il6* |
| *Il1b* | *Il10* |
| *Il1f5* | *Mmp3* |
| *Il1f6* | *Mmp9* |
